# Overlapping Key Populations and HIV Transmission in Tijuana, Mexico: A Modelling Analysis of Epidemic Drivers

**DOI:** 10.1007/s10461-021-03361-2

**Published:** 2021-07-03

**Authors:** Hannah Fraser, Annick Borquez, Jack Stone, Daniela Abramovitz, Kimberly C. Brouwer, David Goodman-Meza, Matthew Hickman, Thomas L. Patterson, Jay Silverman, Laramie Smith, Steffanie A. Strathdee, Natasha K. Martin, Peter Vickerman

**Affiliations:** 1 Oakfield House, Population Health Sciences – Bristol Medical School, University of Bristol, Bristol, BS8 2BN UK; 2 School of Medicine, University of California San Diego, San Diego, USA; 3 David Geffen School of Medicine, University of California Los Angeles, Los Angeles, CA USA

**Keywords:** Mathematical modelling, People who inject drugs, Female sex workers, Men who have sex with men, Mexico, Modelo matemáticopersonas que se inyectan drogas, Mujeres trabajadoras sexuales, Hombres que tienen sexo con hombres, México

## Abstract

Tijuana, Mexico, has a concentrated HIV epidemic among overlapping key populations (KPs) including people who inject drugs (PWID), female sex workers (FSW), their male clients, and men who have sex with men (MSM). We developed a dynamic HIV transmission model among these KPs to determine the extent to which their unmet prevention and treatment needs is driving HIV transmission. Over 2020–2029 we estimated the proportion of new infections acquired in each KP, and the proportion due to their unprotected risk behaviours. We estimate that 43.7% and 55.3% of new infections are among MSM and PWID, respectively, with FSW and their clients making-up < 10% of new infections. Projections suggest 93.8% of new infections over 2020–2029 will be due to unprotected sex between MSM or unsafe injecting drug use. Prioritizing interventions addressing sexual and injecting risks among MSM and PWID are critical to controlling HIV in Tijuana.

**Supplementary Information:**

The online version contains supplementary material available at 10.1007/s10461-021-03361-2.

## Resumen

Tijuana, México, tiene una epidemia de VIH concentrada en poblaciones claves (PC) superpuestas que incluyen personas que se inyectan drogas (PID), trabajadoras sexuales (MTS), sus clientes hombres, y hombres que tienen sexo con hombres (HSH). Desarrollamos un modelo dinámico de transmisión de VIH en estas PC para determinar hasta dónde sus necesidades no atendidas de prevención y tratamiento dirigen la transmisión del VIH. Para 2020–2029 estimamos la proporción de nuevas infecciones adquiridas en cada PC, y la proporción atribuida a sus comportamientos de riesgo sin protección. Estimamos que 43.7% y 55.3% de nuevas infecciones se dan en HSH y PID, respectivamente, con MTS y clientes conformando < 10% de nuevas infecciones. Las proyecciones sugieren que 93.8% de nuevas infecciones en 2020–2029 se deberán a sexo sin protección en HSH o uso inseguro de drogas inyectables. Dar prioridad a intervenciones que atienden los riesgos sexual y de inyección en HSH y PID es crítico para controlar el VIH en Tijuana.

## Introduction

HIV still causes significant global morbidity [1]. Marginalised populations including men who have sex with men (MSM), female sex workers (FSW) and people who inject drugs (PWID) experience the highest disease burden [1]. It is a global priority to quantify the contribution of these key populations (KPs) to on-going HIV transmission and identify interventions to reduce this risk [2–4].

Tijuana, Mexico, is situated on a major drug trafficking route to the US [5]. Sex work is tolerated by police within Tijuana’s *zona roja* [6, 7], with this area also having a thriving drug market [8]. There is also considerable MSM living in Tijuana [9].

The coverage of interventions to reduce HIV transmission among KPs in Tijuana are low. For PWID, the availability of sterile injecting equipment has reduced since support from the Global Fund finished in 2013 [10]. Whilst antiretroviral therapy (ART) coverage is ~ 70% in the general population [11], coverage is much lower among KPs. Similarly, while HIV pre-exposure prophylaxsis (PrEP) effectively prevents HIV acquisition, uptake is low in Tijuana [12].

Mexico has a low adult HIV prevalence (0.54% [9]), but higher prevalence among KPs [11]. In Tijuana, the estimated HIV prevalence in 2012/13 was 2.7% in FSW [13], 17.3% in MSM [14] and 3.5% in PWID [15]. Data also suggests that over one-third of female PWID have recently sold sex [16], a fifth of FSW have ever injected drugs [6], and over one-third of male PWID have ever had sex with men [15], illustrating the overlapping transmission network within Tijuana [17, 18].

While these are useful insights, it is unclear how the unmet treatment and prevention needs of these KPs are contributing to HIV transmission, and therefore how interventions should be targeted. We use HIV transmission modelling to consider this question.

## Methods

### Model Structure

We developed a dynamic HIV transmission model incorporating sexual and injecting HIV transmission among KPs in Tijuana (Supplementary Figures S1&S2).

The female population was stratified into three FSW and PWID groups, while the male population was stratified into seven PWID, MSM and male clients of FSW (denoted as clients) groups (Supplementary Figure S1). Individuals can start one risk behaviour and subsequently initiate another. PWID can cease injecting, FSW and clients can cease selling and buying sex, but MSM remain as MSM.

All individuals enter the model susceptible to HIV. Upon infection, individuals acquire acute HIV infection, before progressing to chronic infection, pre-AIDS, and AIDS [19, 20], whereupon they experience AIDS-related mortality. HIV-infected individuals can initiate (and cease) ART, which reduces disease progression and AIDS-related mortality [21–24]. All individuals experience non-HIV mortality, which is heightened in PWID [25].

HIV transmission occurs among KPs through: heterosexual vaginal and anal sex between men and women from all groups (Supplementary Figure S3); vaginal and anal commercial sex between FSW and clients; anal sex among MSM; and injecting drug use (IDU) among PWID. An individual’s risk of HIV acquisition is related to the HIV prevalence of their sexual or injecting partners. Transmission risk differs for injecting and sexual behaviours [26–28], is elevated if their partners have acute or pre-AIDS infection [19], and is reduced if they are on ART [21–24]. Transmission risk is also related to the frequency and type of sex act for different partnerships, and consistency of condom use. We assume a proportion of heterosexual partnerships are with the general population, and have negligible risk due to their low HIV prevalence. All other partnerships occur between individuals within the modelled population, with random mixing among sub-groups (see Supplementary Materials). We do not model the use of HIV pre-exposure prophylaxis (PrEP) due to its low uptake in Tijuana [12].

### Model Parameterisation

We parameterized our model to Tijuana using data from local studies (Supplementary Table I). Parameters are given in Table 1 and Supplementary Table II.

**Table 1:** Summary of key parameter ranges used for different population groups

	Prior distribution	Distribution	p-value (if applicable)	Data information
(i) Injecting drug use				
Number of injections in the past year among PWID				
Female PWID-FSW; Male PWID; Male PWID-Clients	1440 (IQR: 1080–1440)	Triangular		ECIV
PWID-MSM	RR: 1.3 (95%CI: 1.0–1.7)	Log-normal	0.0369 (χ^2^ test)	ECIV; (RR compared to male PWID)
Female PWID non-FSW	RR: 0.9 (95%CI: 0.8–1.0)	Log-normal	0.0179 (χ^2^ test)	ECIV; (RR compared to male PWID)
Proportion of PWID who have ever shared syringes in the past 6 months				
Female PWID non-FSW; Male PWID only	67.9% (63.3–72.5%)	Truncated normal		ECIV
PWID-client	OR: 14.2 (95%CI: 1.91–104.9)	Log-normal	< 0.0001 (χ^2^ test)	ECIV; (OR compared to male PWID)
PWID-MSM	OR: 3.0 (95%CI: 1.0–10.29)	Log-normal	0. 0691 (χ^2^ test)	ECIV; (OR compared to male PWID)
PWID-FSW	OR: 1.5 (95%CI: 1.0–2.26)	Log-normal	0.0471 (χ^2^ test)	ECIV; (OR compared to male PWID)
Proportion who shared at last injection (if shared in the past 6 months) – same estimate used for all PWID groups	45.9% (38.7–53.2%)	Truncated normal		ECIV
(ii) Men who have sex with men				
Proportion of MSM with at least one main male partner in last year	62.3% (95%CI: 55.4 – 69.2%)	Truncated normal		Proyecto H
Number of main male partners per year (among those that have them in last year)	2 (IQR: 1–2)	Triangular		Proyecto H
Frequency of sex acts in past year with each main partner	25.2 (IQR: 10.2–78)	Triangular		Proyecto H
Consistency of condom use for main partners of MSM	60.8% (52.3–69.2%)	Truncated normal		Proyecto H
Proportion of MSM with at least one casual male partner in last year	60.3% (53.2–67.2%)	Truncated normal		Proyecto H
Number of casual male partners per year (among those that have them in last year)	6 (IQR: 0–24)	Triangular		Proyecto H
Frequency of sex acts in past year with each casual partner	1.1 (IQR: 1–2)	Triangular		Proyecto H
Consistency of condom use for casual partners of MSM	77.9% (71.1–84.8%)	Truncated normal		Proyecto H
(iii) Commercial sex – Female sex workers and their clients				
Number of commercial sex contacts per year				
FSW-PWID	180 (IQR: 72–360)	Triangular		MAPA and MMS
FSW non-PWID	120 (IQR: 60–240)	Triangular		MAPA and MMS
Clients	12 (IQR: 6–24)	Triangular		Sexo Seguro
Consistency of condom use in last commercial sex act among^b^				
Non-PWID FSW reported with clients	85.5% (82.0–89.0%)	Normal		MAPA
PWID FSW reported with clients	RR: 0.8 (95%CI: 0.6–0.9)	Truncated log-normal	< 0.001 (χ^2^ test)	MAPA; (RR compared to non-PWID FSW)
Clients reported with FSW (vaginal sex)	54.8% (48.3–61.3%)	Normal		Sexo Seguro
Clients reported with FSW (anal sex)	45.8% (32.7–58.9%)	Normal		Sexo Seguro
Proportion of commercial sex acts that are vaginal				
FSW with client non-PWID	84.5% (81.7–87.4%)	Truncated normal		Sexo Seguro
FSW with client PWID	RR 0.9 (95%CI: 0.8–1)	Log normal	0.0325 (χ^2^ test)	Sexo Seguro
(iv) Heterosexual main partnerships				
Percentage of each key population with a main partner—included in Supplementary materials as different for each group				
Frequency of vaginal sex acts in past year among				
Female PWID non-FSW with males	48 (IQR: 5–48)	Triangular		ECIV
FSW (PWID and non-PWID) with males	48 (IQR: 20–48)	Triangular		ECIV
Male PWID with females	48 (IQR: 20–48)	Triangular		ECIV
Male Clients with females	60 (IQR: 24–120)	Triangular		Hombre Seguro
MSM with females	24 (IQR: 6–60)	Triangular		Proyecto H
Frequency of anal sex acts in past year among^a^				
All females with males	0 (IQR: 0–5)	Triangular		ECIV
Males (non-MSM) with females	0—6	Uniform		ECIV/Hombre Seguro
MSM with females	6 (IQR: 0.96–19.5)	Triangular		Proyecto H
Consistency of condom use in last sex act for main partnerships				Range over max and min for different surveys
Vaginal sex	8.7–28.3%	Uniform		
Anal sex	4–32.3%	Uniform		
(iv) Heterosexual casual partnerships				
Percentage of each key population with at least one casual partner – included in Supplementary materials as different for each group				
Number of casual partners in past year				
Female PWID non-FSW with males	2 (IQR: 2-4)	Triangular		ECIV
FSW PWID with males	6 (IQR: 4–20)	Triangular		ECIV
Male PWID with females	4 (IQR: 4–6)	Triangular		ECIV
MSM with females	12 (IQR: 6–24)	Triangular		Proyecto H
Clients with females	9 (IQR: 6–12)	Triangular		Sexo Seguro
Frequency of vaginal sex acts in past year among				
Female PWID non-FSW with males	0.75 (IQR: 0–2.5)	Triangular		ECIV
FSW PWID with males	1.25 (IQR: 0.007–8)	Triangular		ECIV
Male PWID with females	0.833 (IQR: 0–6)	Triangular		ECIV
MSM with females	1 (IQR: 1–1.5)	Triangular		Proyecto H
Clients with females	1.3 (IQR: 1–3)	Triangular		Sexo Seguro
Frequency of anal sex acts in past year among^a^				
All females with all males	0 (IQR: 0–0.25)	Triangular		ECIV
All males with all females	0–1	Uniform		Range over male surveys
Consistency of condom use in last sex act for casual partnerships				Take range over all surveys
Vaginal sex	23.3–60.3%	Uniform		
Anal sex	5.2–88.7%	Uniform		

This is included to give a summary with full details of the data used to calibrate the model and prior parameter ranges in the supplementary materials Note that for normal and log-normal distributions the mean and 95% confidence intervals (CI) are given; for triangular distributions the median and interquartile range were used to create the distribution (unless otherwise stated); for uniform distributions a range was taken over the data

^a^To determine frequency of anal sex acts in the past year the rates between males and females are both sampled and then the average of the two is used in the model

^b^Condom use estimates for commercial sex were averaged across reported use by FSW and clients

Using survey data, a PWID’s injecting frequency was generally assumed to be 1,440 (IQR: 1080–1440) per year, but higher for MSM-PWID and lower for non-FSW PWID. Two-thirds (67.9%, 95%CI:63.3–72.5%) of PWID were assumed to share syringes in the last 6 months, although this was heightened among some sub-groups.

Consistency of condom use varies according to KP, partner type and over time, increasing linearly from 1987 [29, 30] up to our survey estimates, with uncertainty being incorporated in the time that this was achieved (up to 10 years earlier).

Based on survey data, nearly two-thirds of MSM were assumed to have main (2 per year, IQR:1–2) and casual male partners (12 per year, IQR:0–48). Commercial sex only occurs between FSW and their male clients, with FSWs having commercial sex every 2–3 days and clients once a month.

ART was assumed to have started in 2003, with coverage being 2–18% amongst PWID and FSW in 2012 [15, 31, 32] and 15–45% amongst MSM and clients in 2017 (unpublished data). Further details of all model parameters are given in the Supplementary Materials.

Priors for the number of individuals in each overlapping group were estimated as follows. We firstly assumed 10,000 PWID [5], with 15.0% (95%CI:12.8–17.1%) of PWID [5] being female. We then estimated the number of FSWs and their overlap with PWID using survey data on injecting behaviours among FSW and commercial sex behaviour among female PWID, estimating 1709 (1620–1826) FSW in 2020 with 477 (443–507) FSW-PWID (Supplementary Figure S4). The client population size was determined through balancing the reported frequency of commercial sex for FSW and clients, giving 8678 clients (7565–10,867). The number of MSM was estimated through different studies, 5276 (4501–6413) (Supplementary Table III).

### Model Calibration

Using an approximate Bayesian computation sequential Monte Carlo (ABC SMC) scheme, accounting for uncertainty in the calibration data and parameters [33], the model was calibrated to our population size estimates; HIV prevalence estimates among all PWID (2005/2007/2012), FSW (2005/2011/2013) and MSM (2012); and ART coverage estimates for PWID (2012), FSW (2012), MSM (2017) and clients (2017) (Supplementary Tables I&II). This scheme starts with 5,000 parameter sets randomly sampled from their prior distributions, which are iteratively perturbed to improve the goodness-of-fit until we achieve our desired criteria, so producing a set of 5,000 baseline model fits (see Supplementary Materials).

### Model Validation

To assess whether the model replicated observed epidemiological trends that it was not calibrated to, the baseline model fits were compared against six HIV incidence estimates among PWID, FSW and clients, and 16 HIV prevalence estimates among PWID and FSW by PWID status. We estimated the average proportion of the 95% confidence intervals (95%CI) for these 22 data estimates that the baseline model fits passed through.

### Impact of Existing Interventions

The model was used to estimate the impact of historical increases in condom use and ART coverage. Compared to counterfactual scenarios where the baseline model fits were run with either no increase in condom use from 1987 or no ART coverage from 2003, we estimated the percentage of infections prevented by either of these intervention until 2020.

### Contribution of Key Populations and Risk Behaviours to new HIV Infections over 2020–2029

For 10-years over 2020–2029, we used the baseline model fits to assess the contribution of each KP and their risk behaviours to new/incident HIV infections occurring among KPs over 2020–2029. This is useful for estimating the best that can be achieved from targeting interventions to different KPs or risk behaviours.

#### Contribution of Different Key Populations

We estimated the proportion of all new infections occurring among each KP over 2020–2029. We then estimated the proportion of new infections that are prevented across all KPs if for each KP in turn, both the risks of becoming infected and infecting others were removed over 2020–2029; equivalent to a perfect intervention making that KP not infectious or susceptible to infection. Although no perfect interventions exist, this impact is likely to be nearly realised with full scale-up of both prevention and treatment interventions such that nearly all transmission is removed.

#### Contribution of Different Risk Behaviours

For each risk behaviour (commercial sex, sex between men, non-commercial heterosexual sex and unsafe IDU), we estimated the proportion of new HIV infections prevented over 2020–2029 if the risk of HIV transmission for each risk behaviour was removed over that time period; equivalent to a perfect intervention making that risk behaviour fully protected. We repeated this for each KP by estimating the proportion of new infections prevented in that KP if their different risk behaviours were fully protected over 2020–2029.

### Sensitivity Analysis

We determined the importance of uncertainty in individual parameters to the overall variability in our estimates for the contribution of different risk behaviours to incident HIV infections over 2020–2029. We performed a linear regression analysis of covariance [34] across our baseline model fits.

Due to uncertainty in the current ART coverage in MSM and clients, we determined how the contribution of different risk behaviours to incident HIV infections would change if the ART coverage in MSM and clients were at national levels for men (70% coverage in 2018). Similarly, because circumstantial evidence suggests that MSM who inject drugs mainly do so with other MSM, we estimated the effect of assuming that MSM-PWID only mix with each other when injecting. We also investigated if the contribution of risk behaviours to incident HIV infections differed for 2010–2019 compared to 2020–2029. Lastly, we investigated the effect of calibrating the model to additional HIV prevalence data among female PWID and FSW-PWID to determine whether this would effect our projections.

## Results

### Model Fit to Data

**Fig. 1:**
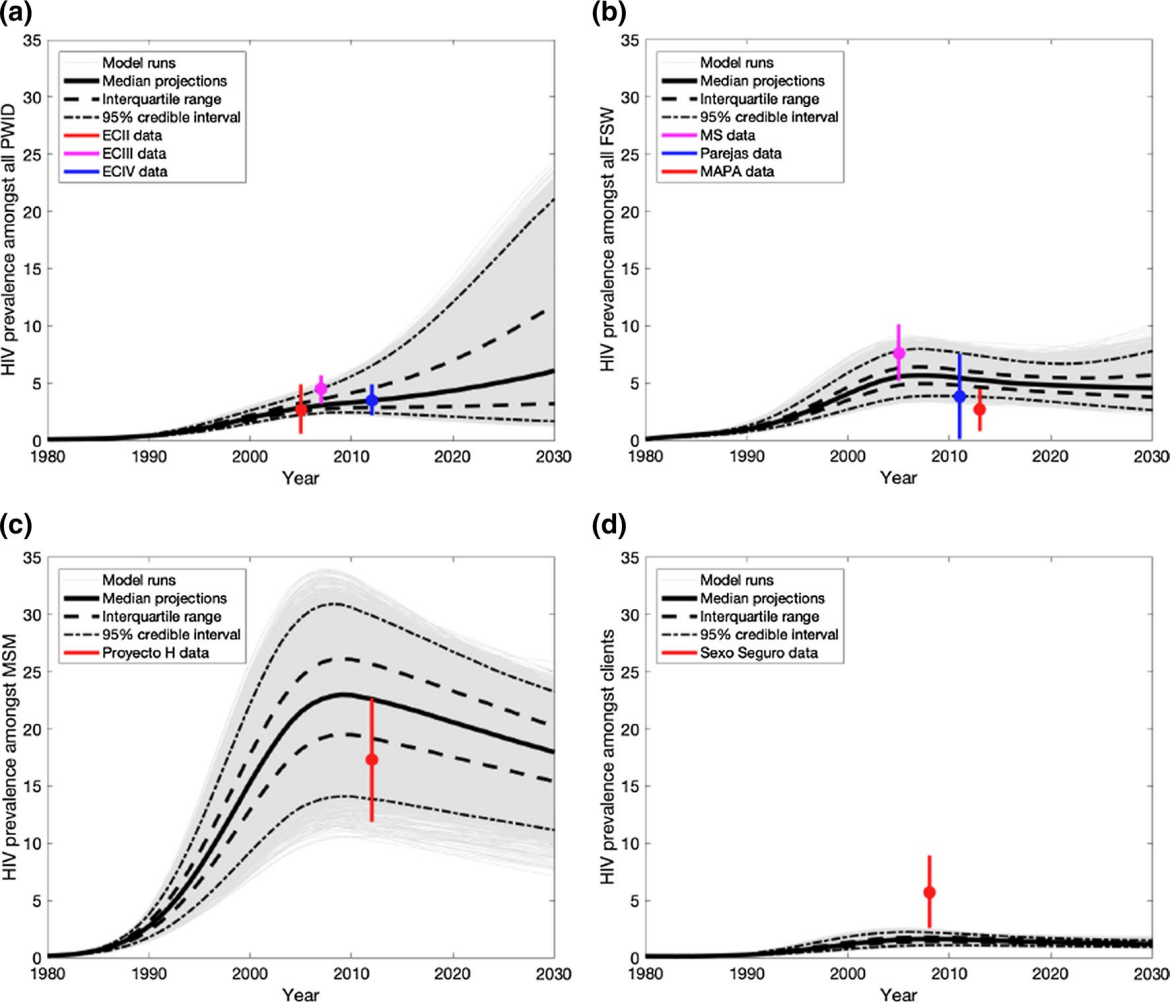
HIV prevalence projections and available data estimates for **a** PWID; **b** FSW; **c** MSM and **d** clients in Tijuana, Mexico. The model was calibrated to HIV prevalence data amongst PWID, FSW and MSM (denoted by circles [mean] and vertical lines [95% confidence intervals]), as well as ART coverage data for all four key populations. Pale grey lines show the model projections for each of the runs, solid black lines show the median of the model runs, dashed black lines show the interquartile range of model runs, and dot-dashed lines show the 95% credibility intervals. Note survey acronyms used in figures: ECII – El Cuete II; ECIII – El Cuete III; EC IV – El Cuete IV; MS – Mujer Segura; MAPA – Salud de MAPA

The baseline model fits reproduced the calibration data (Fig. 1), with HIV prevalence peaking around 2003 among FSW, MSM and clients, but being more uncertain among PWID. The decrease in prevalence post-2003 is due to HIV-related mortality, mainly among MSM, causing a decrease in HIV prevalence in all sub-groups due to their interactions. In 2020, HIV prevalence amongst PWID, FSW, MSM and clients is projected to be 4.4% (95% credible interval (95%CrI):2.1–12.1%), 4.8% (95%CrI:3.4–6.7%), 20.6% (95%CrI:12.7–26.7%) and 1.4% (95%CrI:1.0–1.7%), respectively.

The model validated well against available HIV incidence and prevalence data that it was not calibrated to (Supplementary Figures S6&S7). On average, the 5,000 baseline model fits fell within 85.9% of the 95%CIs for the 6 incidence validation points and 63.9% of the 95%CIs for the 16 prevalence validation points.

### Impact of Existing Interventions

Historic increases in condom use during commercial sex over 1987–2020 have averted 476 (95%CrI:333–753) or 7.8% (95%CrI:5.9–11.3%) of new infections occurring among KPs over that period. Conversely, 5087 (95%CrI:4033–6453) or 47.4% (95%CrI:38.0–58.0%) of new infections occurring among KPs were prevented through increases in condom use among MSM. Lastly, existing ART coverage has prevented 656 (95%CrI:507–862) or 18.5% (95%CrI:12.6–23.0%) of infections occurring among KPs since 2003.

### Contribution of Different Key Populations to HIV Transmission

**Fig. 2:**
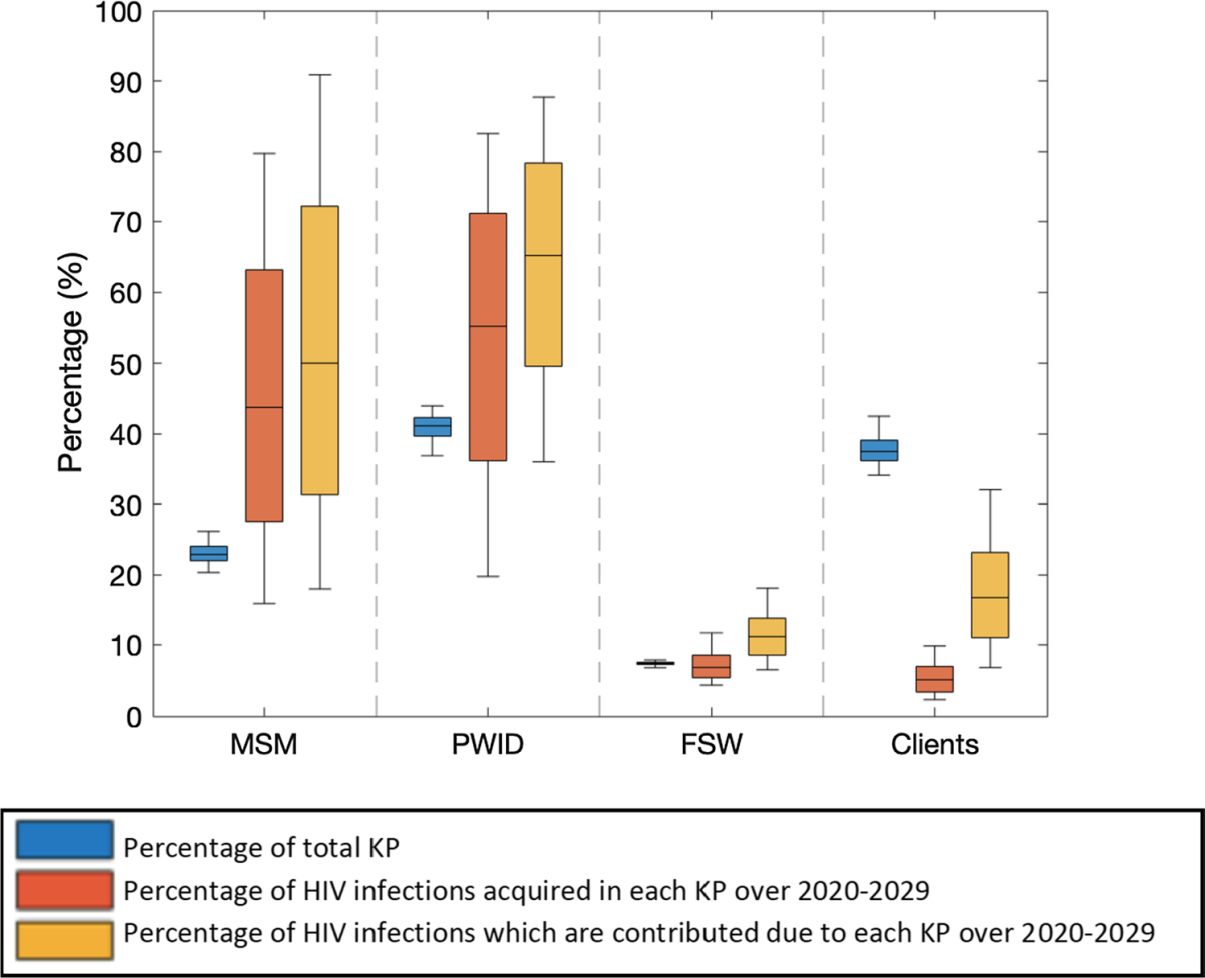
Relative size of each key population, proportion of new infections that occur in each key population and contribution of each key population to HIV transmission over 2020–2029 in Tijuana, Mexico. Blue boxes denote the size of each key population group as a percentage of the total key population size. Orange boxes denote the percentage of new infections occurring within each key population over 2020–2029. Yellow boxes denote the proportion of new HIV infections occurring among KP that will be prevented if all risk behaviours of each key population are fully protected over 2020–2029. Boxes in the figure show the 25th, 50th and 75th percentiles and the whiskers indicate the 2.5th and 97.5th percentiles

Across all KPs, the model projects 1608 (95%CrI:909–3821) new HIV infections over 2020–2029, with most occurring among MSM and PWID and being due to their risk behaviours (Fig. 2). Whilst MSM only make up 22.8% (95%CrI:20.1–26.0%) of all KP in 2020, 43.7% (95%CrI:15.9–79.7%) of new infections occurred among MSM over 2020–2029 and 50.0% (95%CrI:17.9–90.1%) of all new HIV infections occurring among KP will be prevented if all MSM risk behaviours are fully protected. Similarly, PWID comprise 41.1% (95%CrI:36.8–44.0%) of all KP in 2020, with 55.3% (95%CrI:19.7–82.5%) of new infections occurring among PWID over 2020–2029 and 65.2% (95%CrI:36.0–87.7%) of all new HIV infections occurring among KP being prevented if all PWID risk behaviours are fully protected (sexual and injecting risk behaviours). In contrast, FSW and their clients contribute much less to transmission, with 11.2% (95%CrI:6.5–18.1%) and 16.8% (95%CrI:6.8–32.0%) of new HIV infections occurring among KP being prevented if all risk behaviours of FSWs or clients are fully protected, respectively.

### Contribution of Different Risk Behaviours to all HIV Transmission

**Fig. 3:**
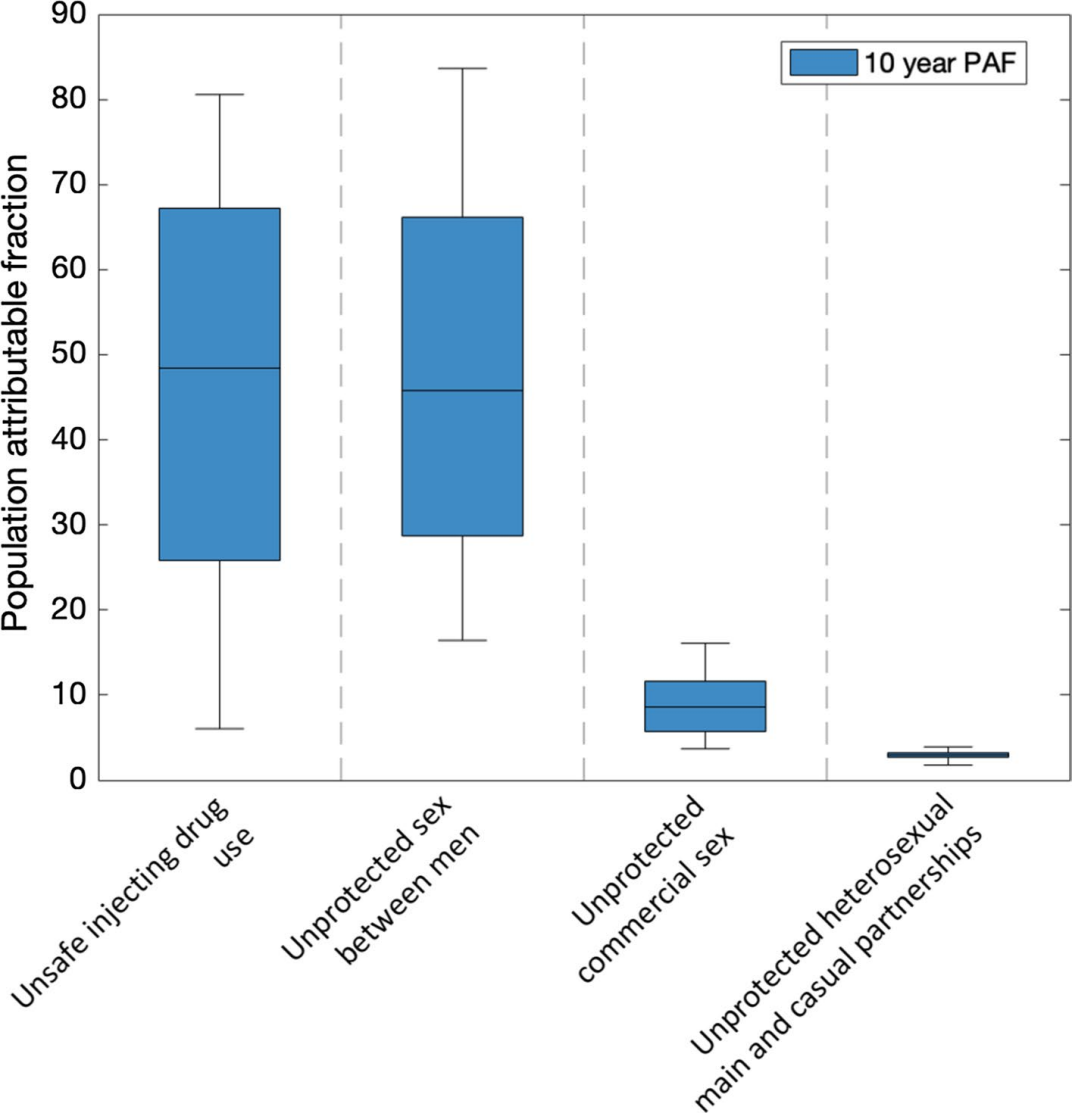
The percentage of new HIV infections occurring among KP that will be prevented if different risk behaviours are fully protected in Tijuana over 2020–2029. Boxes show the 25th, 50th and 75th percentiles and the whiskers indicating the 2.5th-97.5th percentiles over the 5000 baseline model fits. The different scenarios are fully protecting either injecting HIV transmission risk, sex between men, commercial sex; heterosexual sex within main and casual partnerships

Among all KPs, unprotected sex between men and unsafe IDU accounts for most new infections, with 45.8% (95%CrI:16.4–83.6%) and 48.4% (95%CrI:6.0–80.6%) of new HIV infections occurring among KPs being prevented over 2020–2029, respectively, if these risk behaviours are fully protected (Fig. 3). However, the contribution of these risk behaviours are uncertain and inversely related to each other (Supplementary Figure S8), with their overall contribution being high (93.8%; 95%CrI:88.2–97.1%). In contrast, unprotected commercial sex and heterosexual main and casual sex both contribute less than 10% of new HIV infections occurring among KPs over 2020–2029.

### Contribution of Different Risk Behaviours to HIV Transmission for each Key Population

**Fig. 4:**
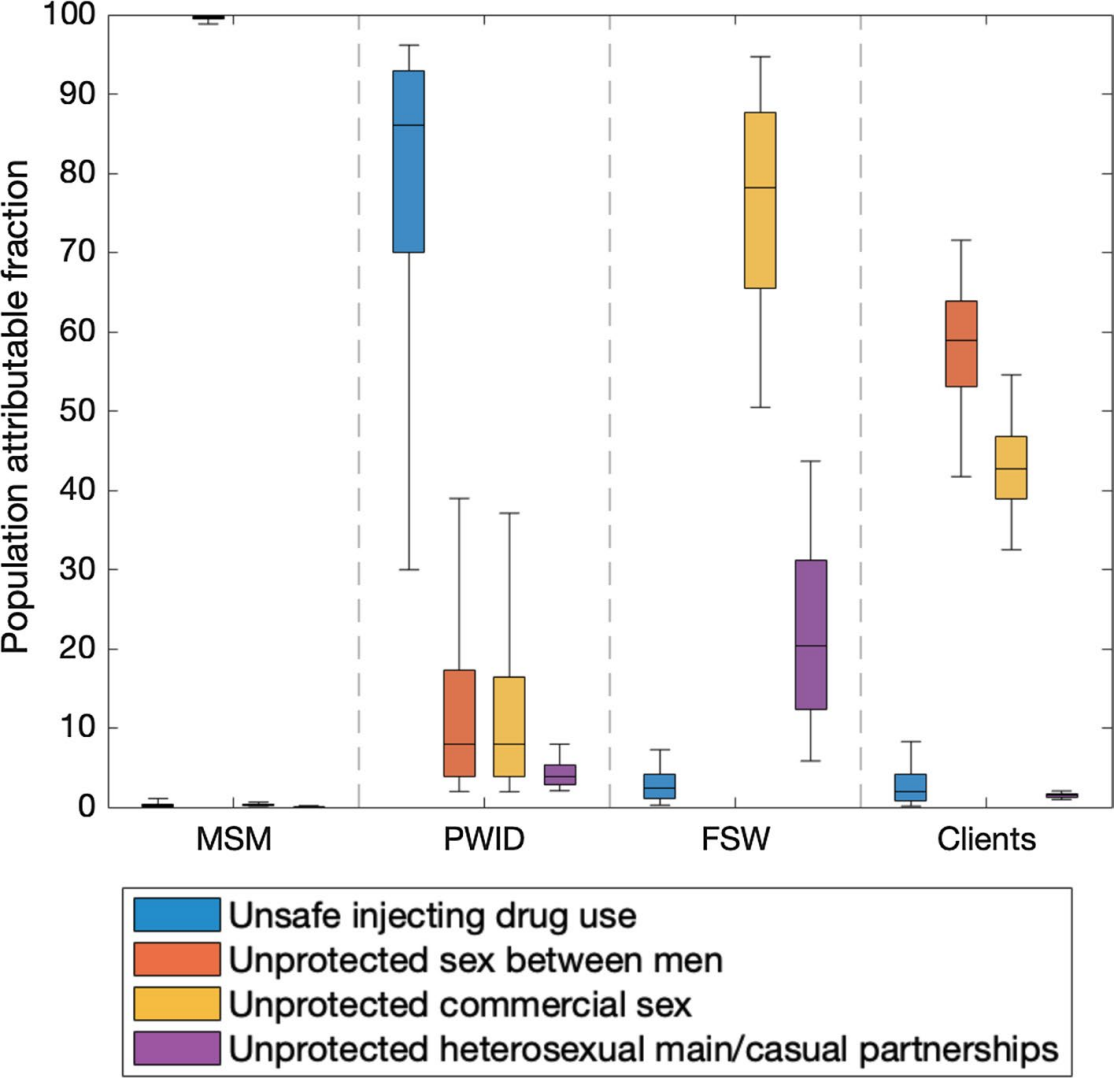
Percentage of new HIV infections occurring in each KP that will be prevented if different risk behaviours are fully protected over 2020–2029 in Tijuana. Boxes show the 25th, 50th and 75th percentiles and the whiskers indicating the 2.5th-97.5th percentiles

For MSM, nearly all (99.7%; 95%CrI:98.8–99.9%) new infections are averted over 2020–2029 if sex between men is fully protected (Fig. 4). Similarly, among PWID, most (86.0%, 95%CrI:30.0–96.2%) new infections are averted when their injecting risk behaviours are protected, although some are also averted (~ 10%) from protecting their sexual risk behaviours. Among FSWs and their clients, most (62.4%; 95%CrI: 49.6–71.0%) new HIV infections over 2020–2029 are averted if commercial sex is fully protected. Much of this is due to commercial anal sex, with 39.7% (95%CrI: 26.9–39.7%) of new infections being prevented if this behaviour is protected. Only a small percentage of infections are averted among FSW and their clients if their injecting risks are protected (2.8%, 95%CrI:0.3–9.4%), while among clients, 58.9% (95%CrI:41.7–71.5%) of new infections are averted if their sex with men is fully protected.

### Sensitivity Analysis

Our ANCOVA analyses indicate that uncertainty in the HIV transmission probability for IDU contributes most to variability in the contribution of unprotected sex between men (95.9%), unsafe IDU (96.4%) and unprotected commercial sex (91.9%) to new HIV infections occurring among KPs over 2020–2029. This variation results in the inverse relationship seen between the contribution of unprotected sex between men and unsafe IDU to new HIV infections (Supplementary Figure S8).

When the modelled HIV prevalence estimates (2020) for PWID, FSW and MSM are included in the ANCOVA, we find that uncertainty in the HIV prevalence of PWID contributes most to the variability in the contribution of unsafe injecting (88.0%), unprotected sex between men (86.2%) and unprotected commercial sex (74.9%) to new HIV infections occurring among KPs. This mirrors what is seen when transmission probabilities are included in the ANCOVA analysis.

If we assume MSM only inject with other MSM (not other PWID), then the contributions of different risk behaviours to new HIV infections are very similar to our baseline findings (Supplementary Figure S9), similar also to what we find if ART coverage is increased among MSM and clients to national levels (70% in 2018).

We also obtain similar results when we consider the contribution of different risk behaviours to new HIV infections over 2010–2019 instead of 2020–2029 (Supplementary Figure S10), with unprotected sex between men and unsafe IDU still accounting for most new infections occurring among KPs, but with unprotected sex between men now being the greater of the two. This also occurs when we calibrate the model to additional prevalence estimates, with unprotected sex between men again accounting for more new infections than unsafe injecting (Supplementary Figure S11).

## Discussion

The overlap of Key populations (KPs) in Tijuana mean multiple HIV transmission routes exist, thereby complicating prevention efforts. We found that over half of on-going HIV transmission occurring among KPs in Tijuana could be prevented if improvements in interventions resulted in sex between men being fully protected, while two-thirds could be prevented if injecting drug use (IDU) was fully protected, indicating that interventions targeting these risk behaviours should be high priority in Tijuana.

Over four-fifths of infections among PWID are associated with unsafe IDU, likely due to minimal harm reduction interventions in Tijuana. This underscores the need to scale-up access to sterile injection equipment, which has become less available since 2013 [10]. Expansion of opioid agonist therapy (OAT), which can reduce the risk of HIV and HCV transmission, and fatal overdose [35, 36], is also needed. Modelling suggests that HIV infections among PWID could also be reduced if Mexico’s policy towards decriminalizing drugs involved linking PWID to OAT instead of incarceration [37]. Importantly, HIV transmission among PWID is also associated with commercial sex and sex between men, indicating that comprehensive interventions addressing sexual risks are also needed.

Among FSW and clients, approximately three-quarters of transmission is due to unprotected commercial sex; therefore addressing this risk remains a high priority. Since sex work is quasi-legal in Tijuana, bars, motels and brothels should provide free access to condoms and promote other prevention approaches such as self-testing for HIV. These approaches should be extended to public sex venues, often frequented by MSM, because they are associated with condomless sex in Tijuana [38]. Previous studies have found that a brief intervention based on motivational interviewing among FSW, including those who injected drugs, is cost-effective in reducing the incidence of HIV and syringe-sharing [39, 40]. Efforts must also focus on structural factors that impede access to HIV prevention and treatment among PWID and sex workers, such as police harassment and abuse [16, 41, 42], with further modelling being needed to project the likely impact of different structural interventions for programme planning.

Despite large gaps in current prevention programming, our findings suggest that existing interventions have had impact. For instance, condom use amongst MSM has prevented nearly half of all HIV infections among KPs in Tijuana since its introduction. In contrast, due to low coverage, ART has only prevented 18.5% of infections since its initiation in 2003. Many challenges hamper access and retention in HIV care among KPs [43, 44] in Tijuana, including the low accessibility of the public HIV clinic [43]. Interventions to scale-up ART are needed to achieve greater coverage and impact, which could include clinics offering integrated services in Tijuana’s *zona roja* accompanied by telemedicine, mobile clinics or peer-navigators to promote engagement in HIV care and ART adherence. A recent modelling analysis [45] found that integrating ART and scaling up OAT in Tijuana could provide synergistic benefits through OAT enhancing ART uptake and retention, preventing HIV infections and overdose deaths, whereas the city’s reliance on compulsory abstinence programmes could cause harm [45]. PrEP is highly effective among MSM in high-income countries [46], and can be highly effective among males and females in LMICs [47–49]. New longer acting PrEP options also show promise [50]. However, a recent study in Tijuana found that of those MSM aware of PrEP, only 4.8% used PrEP [12]. Studies among MSM and FSW in Tijuana have indicated willingness to use PrEP, however perceived barriers to entry including costs and limited access need to be addressed [12, 51, 52].

### Strengths and Limitations

The main strength of our analysis is the wealth of behavioural and epidemiological data from Tijuana used to parameterise and calibrate our model, which captures HIV transmission from overlapping risk behaviours among KPs.

Limitations include the difficulty in estimating the sizes of marginalised populations [53, 54]. Indeed, whilst population size estimates for PWID (6400–10,000) [55], FSW (4850–9000[55]) and MSM (12,227–20,378[9]) exist for Tijuana, these are old and were not based on reliable methods. Therefore, we estimated the population sizes of each KP by using survey data on the overlap of risk groups. This resulted in lower estimates for the number of FSW (1709, 95%CrI:1620–1826) and MSM (5276, 95%CrI:4501–6413). Although the overall number of KPs could be greater, their relative sizes will still need to remain consistent with each other as suggested by survey data. This means that the relative contribution of different risk behaviours should remain robust even if the estimated number of infections are rescaled for different KP sizes.

Secondly, although the model fit well to available HIV prevalence and ART coverage data, and was validated against 22 other prevalence and incidence data points, it was not able to fit all available data. Specifically, the projected HIV prevalence among clients was less than observed in surveys [18], meaning we may have underestimated the contribution of clients to HIV transmission. However, the survey used to estimate the HIV prevalence among clients is thought to have captured higher risk clients with higher HIV prevalence because many reported unprotected commercial sex and IDU [18]. Additionally, there was only one HIV prevalence estimate and no incidence data for MSM. Further studies are needed to better calibrate the MSM aspect of the model to confirm our findings that MSM contribute substantially to HIV transmission in Tijuana.

Thirdly, few estimates of ART coverage exist among KPs, with these being based on self-report. Reassuringly, sensitivity analyses suggest our results were not affected by this uncertainty.

### Comparison with Existing Literature

A wealth of HIV modelling among KPs exists for other settings. While other models [56–59] have investigated HIV dynamics among multiple KPs, none have incorporated overlapping risk behaviours as we have. Other models have also incorporated heterogeneity in risk, but have focussed on fewer KPs [60–63] or just sexual HIV transmission [64–66]. Some models have estimated how unprotected risk behaviours of different KPs [67, 68] affect HIV transmission, highlighting the interventions required to reduce transmission risk, but have generally not considered how the overlapping dynamics of KPs may impact this. In settings like Tijuana, where multiple risk behaviours are highly prevalent, more granular models are needed to provide detailed insights. To date, HIV transmission models for Tijuana have focused on the cost-effectiveness of scaling-up interventions among particular KPs [40, 69] or modelling drug law reform among PWID [37, 41]. This study presents the first dynamic HIV transmission model among all main KPs in Tijuana.

## Conclusions

Settings with overlapping KPs and multiple HIV risk behaviours will require multi-layered combined approaches to provide effective and efficient HIV prevention and treatment efforts. Our modelling indicates that prioritizing interventions to address sexual risk behaviours among MSM, and both sexual and injecting risks among PWID is critical to controlling the HIV epidemic in Tijuana. While some interventions need to be targeted at specific KPs, others are important for all KPs. For example, the coverage of harm reduction interventions is currently very low, and should be prioritised among PWID, while PrEP, which reduces HIV acquisition risk, should be introduced in all groups. In contrast, low ART coverage levels among all KPs highlight the urgent need for targeted scale-up to reduce mortality and prevent onwards HIV transmission among these groups. Our results suggest that reducing HIV acquisition (as PrEP does) and preventing onwards transmission (as ART does) could have a large impact on the number of infections occurring over the next 10 years, indicating the importance of scaling-up interventions. To aid the prioritisation of this improvement in HIV programming, cost-effectiveness analyses are needed to identify the most efficient strategies to reduce HIV incidence in Tijuana.

## Supplementary Information

Below is the link to the electronic supplementary material.

Supplementary file1 (DOCX 5279 KB)

## Data Availability

Study data is available upon request.

## Code Availability

Model code is available upon request.
