# Supplementary material for: Overlapping Key Populations and HIV Transmission in Tijuana, Mexico: A Modelling Analysis of Epidemic Drivers
Source: AIDS Behav. 2021 Jul 3;25(11):3814–27. doi: 10.1007/s10461-021-03361-2 (PMC8560668; doi:10.1007/s10461-021-03361-2)
Supplement: Supplementary file 1 — Supplementary file1 (DOCX 5279 KB) [file 10461_2021_3361_MOESM1_ESM.docx]

# Supplementary Materials: Overlapping high-risk groups and HIV transmission in Tijuana, Mexico: a modelling analysis of epidemic drivers

Contents

[Supplementary Materials: Overlapping high-risk groups and HIV transmission in Tijuana, Mexico: a modelling analysis of epidemic drivers 1](#_Toc55900442)

[Methods 2](#_Toc55900443)

[Mathematical model 2](#_Toc55900444)

[Model equations 4](#_Toc55900445)

[Determining the number of partners for heterosexual transmission 9](#_Toc55900446)

[Model setting and main data 11](#_Toc55900447)

[Model parameterisation 13](#_Toc55900448)

[Model calibration 17](#_Toc55900449)

[Log-likelihood calculation 17](#_Toc55900450)

[OST and NSP coverage in Tijuana 18](#_Toc55900451)

[Results 18](#_Toc55900452)

[Model calibration – ART coverage 18](#_Toc55900453)

[Model validation 19](#_Toc55900454)

[HIV incidence 19](#_Toc55900455)

[HIV prevalence by groups 20](#_Toc55900456)

[Contribution of different risk behaviours to HIV transmission 21](#_Toc55900457)

[Sensitivity analysis 21](#_Toc55900458)

[References 22](#_Toc55900459)

# Methods

## Mathematical model

We developed a dynamic, deterministic, overlapping HIV transmission model incorporating sexual and injecting HIV transmission among key populations in Tijuana, Mexico. The model was stratified by gender, key population (people who inject drugs(PWID), men who have sex with men (MSM), female sex workers (FSW) and their clients, and overlapping groups; Supplementary Figure 1), HIV infection state (susceptible, acute infection, chronic infection, pre-AIDS and AIDS) and HIV treatment status (on/off antiretroviral therapy (ART)).

**Supplementary Figure S1:** Schematic showing the transitions of individuals between different key populations; (a) shows movement between female key populations, and (b) shows movement between male key populations. Key female risk populations are people who inject drugs (PWID) and female sex workers (FSW), while for the male population key risk populations are people who inject drugs, clients of female sex workers (clients) and men who have sex with men (MSM).

(a)


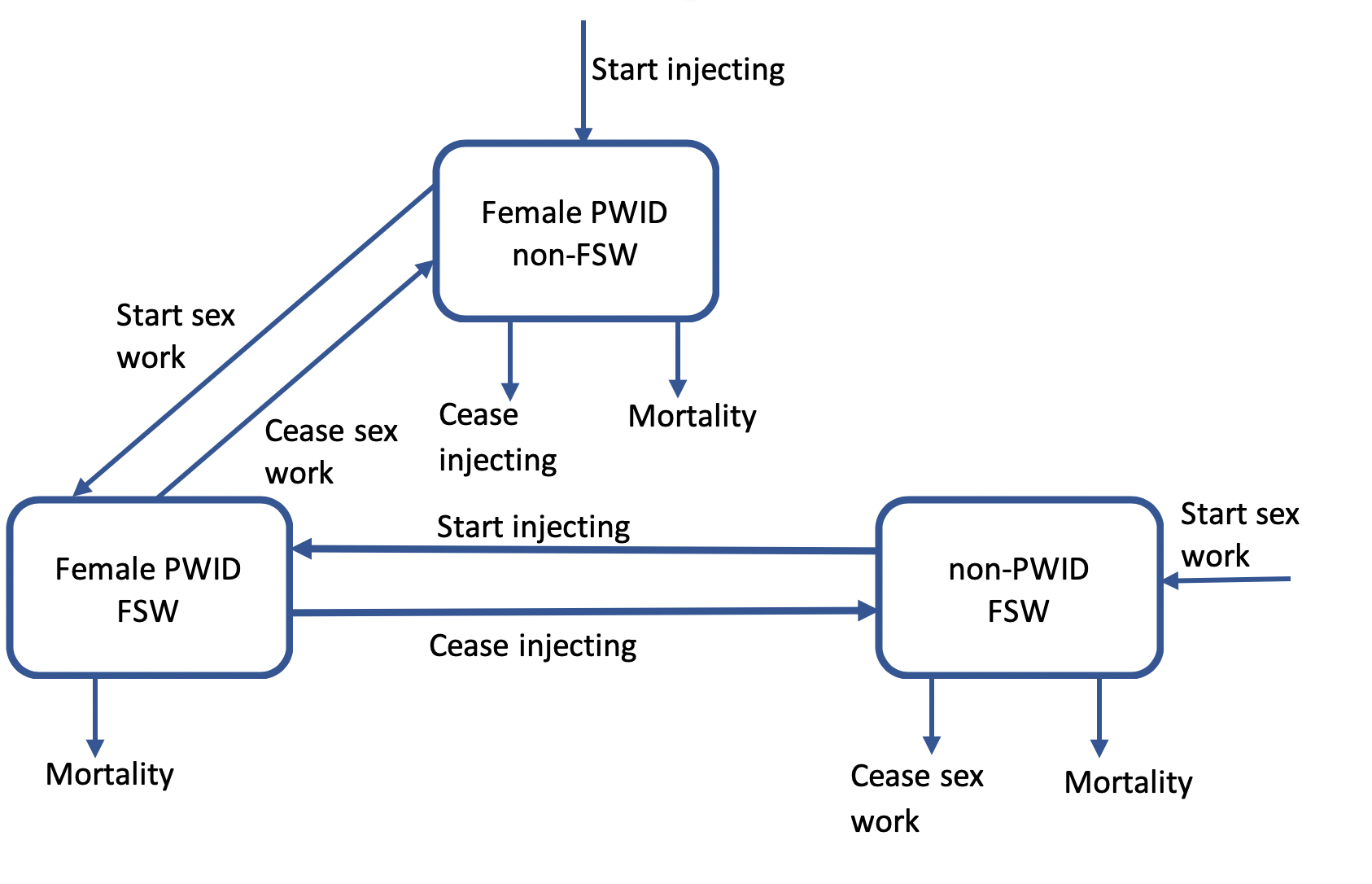


(b)


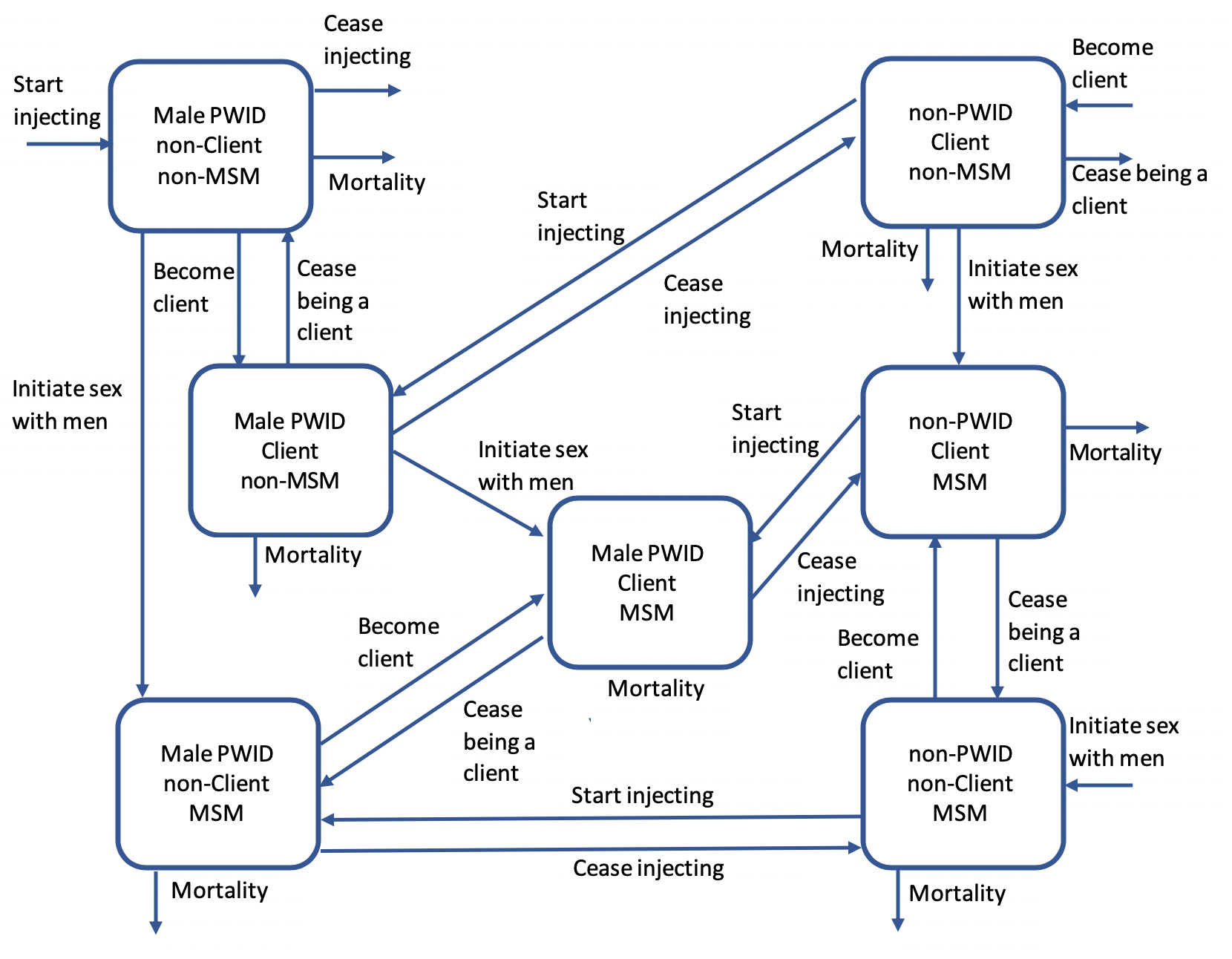


The model stratifies the population with respect to HIV infection and disease progression (susceptible, acute infection, chronic infection, pre-AIDS, AIDS, and on ART, Supplementary Figure S2). Due to low general population prevalence (0.54%)^1^, we assume that all individuals enter the model as susceptible to HIV. Susceptible individuals become infected, with different risks attributed to injecting and sexual (both vaginal and anal) transmission^2-4^. Once infected, individuals experience acute infection characterised by an increased risk of transmission, before progression to chronic infection^5^, then the pre-AIDS phase where there is an increased risk of disease transmission^6^ and finally to the AIDS compartment before AIDS-related mortality occurs^5^. We assume that individuals in the AIDS phase of infection not on ART do not contribute to transmission. Individuals in the chronic infection, pre-AIDS and AIDS phases can be recruited onto ART. Once on ART, progression through disease stages is slowed by 70-80%^7-10^. We assume individuals on ART can be lost to follow-up^11^, whereupon they return to the corresponding infection stage where they can be re-recruited back on to ART. Individuals in the AIDS phase of infection on ART have the same infectivity as those in the pre-AIDS phase of infection on ART. All groups experience non-HIV related mortality, with PWID experiencing a higher mortality rate including drug-related deaths.

**Supplementary Figure S2:** Model schematic for HIV infection and ART status. Note that only individuals who are either chronically HIV infected, have pre-AIDS or AIDS can be recruited on to ART.

The model captures HIV transmission among the different groups through vaginal and anal sex between male and female groups, anal sex within the MSM group, and injecting within the PWID groups. We incorporate heterosexual vaginal and anal sex due to main and casual partnerships and transactions from sex work. Transactions from sex work occur between FSW and their clients and all other heterosexual main and casual partnerships occur between all groups. Main and casual partnerships between men only occur among MSM groups, and we do not distinguish between insertive and receptive sex acts. The risk of HIV transmission for an individual is related to the HIV prevalence of their sexual partners, with transmission risk elevated if they are in the acute or pre-AIDS phases of infection and reduced if they are on ART. Transmission risk is also related to frequency of sex acts for different types of partnerships, the proportion of sex acts that are vaginal/anal and consistency of condom use, which varies according to partner type. Furthermore, risk of transmission differs according to type of sex act (vaginal or anal). We assume random mixing among all MSM (irrespective of group), while the number of heterosexual main and casual partnerships are balanced within the model. We assume that only a proportion of heterosexual main and casual partnerships occur within the model (Main paper –Table I) based on the proportion of individuals who had such type of partnership with a particular group. Due to low general population prevalence we did not include heterosexual main and casual partnerships with the general population. We assume random injecting-related mixing between male and female PWID (including all PWID groups overlapping with other groups).

## Model equations

Let $X_{i,j}$ be the number of individuals in the model where

- Subscript $i$ denotes group ($i=1$: female PWID only; $i=2$: female PWID FSW; $i=3$: FSW only; $i=4$: male PWID only; $i=5$: male PWID client; $i=6$: male PWID MSM; $i=7$: male PWID client MSM; $i=8$: male client only; $i=9$: male client MSM; $i=10$: MSM only).
- Subscript $j$ denotes HIV infection stage ($j=1$: susceptible; $j=2$: acute infection; $j=3$: chronic infection; $j=4$: pre-AIDS phase; $j=5$: AIDS phase; $j=6$: chronic infection on ART; $j=7:$ pre-AIDS phase on ART; $j=8$: AIDS phase on ART.

The ordinary differential equation models can be written as

$$\frac{dX_{i,j}}{dt}=\Theta_{i,j}+\Sigma_{i,j}+\Lambda_{i,j}+\Pi_{i,j}+M_{i,j}$$

where

- $\Theta_{i,j}$ denotes the inflow into different population groups
- $\Sigma_{i,j}$ denotes the transitions between different population sub-groups within the model
- $\Lambda_{i,j}$ denotes HIV transmission within the model
- $\Pi_{i,j}$ denotes HIV disease progression and transitions on/off ART
- $M_{i,j}$ denotes mortality

**Inflow into different groups**

$\Theta_{i,j}$ denotes the inflow of individuals into each of the groups and is given by

$$\left\{ \begin{aligned} \Theta_{i,j}=0 i=2,5,6,7,9;\forall j \\ \Theta_{i,1}=\bar{\alpha}_{i}Y_{i} i=1,4 \\ \Theta_{i,1}=\bar{\kappa}_{i}Y_{i} i=3 \\ \Theta_{i,1}=\bar{z}_{i}Y_{i} i=8 \\ \Theta_{i,1}=\chi Y_{i} i=10 \end{aligned} \right.$$

where $Y_{i}$ is the number of males/females who would be eligible to enter sub-group $i$ based on the population size and age structure in Tijuana, and

- $\bar{\alpha}_{i}$ denotes the rate individuals initiate injecting into group $i$
- $\bar{\kappa}_{i}$ denotes the rate women initiate sex work into group $i$
- $\bar{z}_{i}$ denotes the rate men initiate buying sex into group $i$
- $\chi$ denotes the rate that men transition into the MSM group.

**Transitions between population sub-groups**

These terms are concerned with movement between different sub-groups (i.e. initiating/ceasing different risk behaviours)

$$\Sigma_{1,j}=-\left( \kappa_{1}+\sigma_{FN} \right)X_{1,j}+\gamma_{2}X_{2,j}$$

$$\Sigma_{2,j}=\kappa_{1}X_{1,j}-\left( \gamma_{2}+\sigma_{FF} \right)X_{2,j}+\alpha_{F}X_{3,j}$$

$$\Sigma_{3,j}=\sigma_{FF}X_{2,j}-\left( \gamma_{3}+\alpha_{F} \right)X_{3,j}$$

$$\Sigma_{4,j}=-\left( \chi+\sigma_{M}+z_{4} \right)X_{4,j}+\phi_{I}X_{5,j}$$

$$\Sigma_{5,j}=z_{4}X_{4,j}-\left( \chi+\phi_{I}+\sigma_{M} \right)X_{5,j}+\alpha_{M}X_{8,j}$$

$$\Sigma_{6,j}=\chi X_{4,j}-\left( \sigma_{M}+z_{6} \right)X_{6,j}+\phi_{I}X_{7,j}+\alpha_{M}X_{10,j}$$

$$\Sigma_{7,j}=\chi X_{5,j}+z_{6}X_{6,j}-\left( \phi_{I}+\sigma_{M} \right)X_{7,j}+\alpha_{M}X_{9,j}$$

$$\Sigma_{8,j}=\sigma_{M}X_{5,j}-\left( \alpha_{M}+\chi+\phi_{N} \right)X_{8,j}$$

$$\Sigma_{9,j}=\sigma_{M}X_{7,j}+\chi X_{8,j}-\left( \alpha_{M}+\phi_{N} \right)X_{9,j}+z_{10}X_{10,j}$$

$$\Sigma_{10,j}=\sigma_{M}X_{6,j}+\phi_{N}X_{9,j}-\left( \alpha_{M}+z_{10} \right)X_{10,j}$$

where

- $\kappa_{i}$ denotes the rate of initiating sex work for individuals in group $i (i=1)$
- $\sigma_{k}$ denotes the rate of cessation of injecting for individuals with subscripts denoting: $FN$: female PWID non-FSW; $FF$: female PWID FSW; $M$: male PWID.
- $1/\gamma_{i}$ denotes the average duration of sex work for FSW in group $i (i=2,3)$
- $\alpha_{k}$ denotes the rate of initiating injecting for individuals in group with subscripts denoting: $F$: females; $M$: males. Note that these rates of initiating injecting within the model are different to those outside the model.
- ${1/\phi}_{k}$denotes the average duration of buying sex for individuals with subscripts denoting: $I$: clients who also inject drugs; $N$: clients who do not inject drugs.
- $\chi$ denotes the rate that males transition into the MSM group
- $z_{i}$ denotes the rate of initiating buying sex for individuals in group $i (i=4,6,10)$.

**HIV transmission**

$\Lambda_{i,j}$ describes HIV transmission

$$\Lambda_{i,1}=-(\lambda_{i}^{inj}+\lambda_{i}^{sex})X_{i,1}$$

$$\Lambda_{i,2}=\left( \lambda_{i}^{inj}+\lambda_{i}^{sex} \right)X_{i,1}$$

$$\Lambda_{i,j}=0 j=3,\ldots,8$$

where

- $\lambda_{i}^{inj}$ denotes the HIV injecting force of infection among PWID ($i=1,2,4,5,6,7$; see section below)
- $\lambda_{i}^{sex}$ denotes the HIV sexual force of infection in group $i$ (see section below)

HIV injecting force of infection

The HIV injecting force of infection for individuals in group $i=1,2,4,5,6,7$ (i.e. PWID sub-groups) is denoted by $\lambda_{i}^{inj}$ and is given by

$$\lambda_{i}^{inj}=\beta_{inj}m_{i}\sum_{k=1,2,4,5,6,7} \rho_{k}^{inj}\frac{Q_{k}^{inj}}{N_{k}}$$

where

$$\rho_{i}^{inj}=\frac{m_{i}N_{i}}{\sum_{k=1,2,4,5,6,7} m_{k}N_{k}}$$

$$N_{i}=\sum_{j} X_{i,j}$$

$$Q_{i}^{inj}=\epsilon_{A}X_{i,2}+X_{i,3}+\epsilon_{P}X_{i,4}+\left( 1-\epsilon_{T} \right)X_{i,6}+\epsilon_{P}(1-\epsilon_{T})(X_{i,7}+X_{i,8})$$

and

- $\beta_{inj}$ denotes the HIV transmission rate for injecting for PWID
- $m_{i}$ denotes the frequency of injecting per year among PWID in group $i$
- $\rho_{i}^{inj}$ is the proportion of injecting contacts among group $i$.
- $N_{i}$ denotes the total number of PWID in group $i$ (see equation below)
- $\epsilon_{A}$ denotes the relative increase in HIV transmissibility if in the acute stage of infection compared to the chronic stage of infection
- $\epsilon_{P}$ denotes the relative increase in HIV transmissibility of in the pre-AIDS phase of infection compared to the chronic stage of infection
- $(1-\epsilon_{T})$ denotes the relative reduction in disease progression due to ART.

For all other sub-groups $(i=3,8,9,10)$ $\lambda_{i}^{inj}=0$.

HIV sexual force of infection

The HIV sexual force of infection has several components and is denoted by

$$\lambda_{i}^{sex}=\lambda_{i}^{MSM-M}+\lambda_{i}^{MSM-L}+\lambda_{i}^{C}+\lambda_{i}^{M}+\lambda_{i}^{L}$$

where

- $\lambda_{i}^{MSM-M}$ denotes sexual transmission from main partnerships between MSM (groups $i=6,7,9,10$ only)
- $\lambda_{i}^{MSM-L}$ denotes sexual transmission from casual partnerships between MSM (groups $i=6,7,9,10$ only)
- $\lambda_{i}^{C}$ denotes commercial sexual transmission between FSW and their clients (groups $i=2,3,5,7,8,9$ only).
- $\lambda_{i}^{M}$ denotes heterosexual sexual transmission through main partnerships between all groups.
- $\lambda_{i}^{L}$ denotes heterosexual sexual transmission through casual partnerships between all groups.

a. Sexual transmission between MSM (main and casual partnerships)

The HIV sexual force of transmission for partnerships type $MSM-Y$ where $Y=M$ (main partnerhips) or $Y=L$ (casual partnerships)

$$\lambda_{i}^{MSM-Y}=\sum_{j} \frac{\left( \beta^{A_{RE}}+\beta^{A_{IN}} \right)}{2}d_{i}^{MSM-Y}\sum_{k=6,7,9,10} \left( 1-\epsilon P_{k,j}^{MSM-Y} \right)\psi_{k,j}^{MSM-Y}\rho_{k}^{MSM}\frac{Q_{k}}{N_{k}}$$

where

$$\rho_{i}^{MSM-Y}=\frac{d_{i}^{MSM-Y}N_{i}}{\sum_{k=6,7,9,10} d_{k}^{MSM-Y}N_{k}}$$

$$P_{i,j}^{MSM-Y}=\frac{\hat{P}_{i,j}^{MSM-Y}+\hat{P}_{j,i}^{MSM-Y}}{2}$$

and

- $\beta^{A_{RE}}$and $\beta^{A_{IN}}$ denote the HIV transmission rates for receptive and insertive anal sex, respectively and are divided by two as the model does not distinguish between receptive and insertive sexual acts in the force of infection.
- $d_{i}^{MSM-Y}$ is the total number of partnerships of type $Y$ that an individual in group $i$has per year.
- $\epsilon$ is the efficacy of condom use
- $P_{i,j}^{MSM-Y}$ denotes the average frequency of condom use between groups $i$ and $j$ (i.e. $\hat{P}_{i,j}^{MSM-Y}$ is the frequency of condom use of group $i$with group $j$ and $\hat{P}_{j,i}^{MSM-Y}$ is the frequency of condom use of group $j$ with group $i$.
- $\psi_{i,j}^{MSM-Y}$ is the average number of sex acts of type $Y$ between group $i$ and group $j.$ This is calculated by taking the average between groups $i$ and $j$ such that $\psi_{i,j}^{MSM-Y}=\psi_{j,i}^{MSM-Y}$
- $\rho_{i}^{MSM-Y}$ is the proportion of individuals in group $i$ who have partnerships of type $Y$
- $\frac{Q_{i}}{N_{i}}$ as defined previously.

b. Commercial sexual transmission between FSW and their clients

The HIV sexual forces of transmission for commercial partnerships among FSW $(i=2,3)$ and the clients $(i=5,7,8,9)$ are given by:

For FSW $(i=2,3)$

$$\lambda_{i}^{C}=\sum_{j} \bar{n}_{i}\sum_{k=5,7,8,9} {[\beta}^{V_{MF}}\left( 1-\epsilon P_{V,i,j}^{C} \right)\Psi_{V,i,k}^{C}+\beta^{A_{RE}}\left( 1-\epsilon P_{A,i,j}^{C} \right)\Psi_{A,i,k}^{C}]\rho_{k}^{C-Cli}\frac{Q_{k}}{N_{k}}$$

where

$$\rho_{i}^{C-Cli}=\frac{n_{i}^{C}N_{i}}{\sum_{k=5,7,8,9} n_{k}^{C}N_{k}} i=5,7,8,9$$

and

- $\bar{n}_{i} (i=2,3)$ is the adjusted number of commercial partners that FSW have with clients (see section on Determining the number of partners for heterosexual transmission)
- $\beta^{V_{MF}}$ denotes the HIV transmission rate for vaginal sex from men to women
- $P_{V,i,j}^{C}$ and $P_{A,i,j}^{C}$ denote the average frequency of condom use for vaginal and anal commercial sex between groups $i$ and $j$
- $\Psi_{V,i,j}^{C}$ and $\Psi_{A,i,j}^{C}$ denote the proportion of commercial sex acts that are vaginal and anal between groups $i$ and$j$, respectively.
- $n_{i}$is the number of commercial partners that an individual in group $i$ has each year

For clients $(i=5,7,8,9)$:

$$\lambda_{i}^{C}=\sum_{j} n_{i}\sum_{k=2,3} {[\beta}^{V_{FM}}\left( 1-\epsilon P_{V,i,k}^{C} \right)\Psi_{V,i,k}^{C}+\beta^{A_{IN}}\left( 1-\epsilon P_{A,i,k}^{C} \right)\Psi_{A,i,k}^{C}]\rho_{k}^{C-FSW}\frac{Q_{k}}{N_{k}}$$

where

$$\rho_{i}^{C-FSW}=\frac{n_{i}^{C}N_{i}}{\sum_{k=2,3} n_{k}^{C}N_{k}} i=2,3$$

and

- $\beta^{V_{FM}}$ denotes the HIV transmission rate for vaginal sex from women to men.

All other parameters have been previously defined.

c. Heterosexual main and casual partnerships between all sub-groups

The HIV sexual forces of transmission for partnership type $Y$ ($Y=M$ main partnerhips; $Y=L$ – casual partnerships) among women ($i=1,2,3)$ and men $(i=4,\ldots,7)$ are given by:

For women $(i=1,2,3)$:

$$\lambda_{i}^{Y}=\sum_{j} r_{i}^{Y}\sum_{k=4,\ldots,10} {[\beta}^{V_{MF}}\left( 1-\epsilon P_{V,i,k}^{Y} \right)\Psi_{V,i,k}^{Y}+\beta^{A_{RE}}\left( 1-\epsilon P_{A,i,k})\Psi_{A,i,k}^{Y} \right]\rho_{k}^{Y-M}\frac{Q_{k}}{N_{k}}$$

where

$$\rho_{i}^{Y-M}=\frac{r_{i}^{Y}N_{i}}{\sum_{k=4,\ldots,10} r_{k}^{Y}N_{k}}$$

And for men $(i=4,\ldots,10)$

$$\lambda_{i}^{Y}=\sum_{j} \bar{r}_{i}^{Y}\sum_{k=1,2,3} {[\beta}^{V_{FM}}\left( 1-\epsilon P_{V,i,k}^{Y} \right)\Psi_{V,i,k}^{Y}+\beta^{A_{IN}}\left( 1-\epsilon P_{A,i,k})\Psi_{A,i,k}^{Y} \right]\rho_{k}^{Y-F}\frac{Q_{k}}{N_{k}}$$

where

$$\rho_{i}^{Y-F}=\frac{r_{i}^{Y}N_{i}}{\sum_{k=1,2,3} r_{k}^{Y}N_{k}}$$

and

- $r_{i}^{Y}$ is the reported number of partnerships of type $Y$ that an individual in group $i$has in a year within the model.
- $\bar{r}_{i}^{Y}$ is the adjusted number of partnerships of type $Y$ that an individual in group $i$has in a year within the model.

and all other parameters previously defined.

**HIV disease progression**

$\Pi_{i,j}$ describes transitions between different infection stages, as well as transitions on and off ART.

$$\Pi_{i,1}=0$$

$$\Pi_{i,2}=-\pi_{E}X_{i,2}$$

$$\Pi_{i,3}=\pi_{E}X_{i,2}-\pi_{C}X_{i,3}-\omega_{i}X_{i,3}+l_{i}X_{i,6}$$

$$\Pi_{i,4}=\pi_{C}X_{i,3}-\pi_{P}X_{i,4}-\omega_{i}X_{i,4}+l_{i}X_{i,7}$$

$$\Pi_{i,5}=\pi_{P}X_{i,4}-\pi_{A}X_{i,5}-\omega_{i}X_{i,5}+l_{i}X_{i,8}$$

$$\Pi_{i,6}=-\delta\pi_{C}X_{i,6}+\omega_{i}X_{i,3}-l_{i}X_{i,6}$$

$$\Pi_{i,7}=\delta\pi_{C}X_{i,6}-\delta\pi_{p}X_{i,7}+\omega_{i}X_{i,4}-l_{i}X_{i,7}$$

$$\Pi_{i,8}=\delta\pi_{P}X_{i,7}-\delta\pi_{A}X_{i,8}+\omega_{i}X_{i,5}-l_{i}X_{i,8}$$

where

- $\pi_{E}$ is the rate of progressing from acute HIV infection to chronic HIV infection
- $\pi_{C}$ is the rate of progressing from chronic HIV infection to pre-AIDS phase of infection
- $\pi_{P}$ is the rate of progression from pre-AIDS phase of infection to AIDS phase of infection
- $\pi_{A}$ is the HIV-related mortality associated with being in the AIDS phase of infection
- $\delta$ is the relative reduction in disease progression and mortality associated with being on ART
- $\omega_{i}$ is the rate of enrolling onto ART in group $i$
- $l_{i}$ is the loss to follow-up from ART in group $i$.

**Mortality**

$M_{i,j}$ represents non-HIV related mortality within the model.

$$\left\{ \begin{matrix} M_{i,j}=\mu_{i} i=3,8,9,10 \\ M_{i,j}=\bar{\mu}_{i} i=1,2,4,5,6,7 \end{matrix} \right.$$

where

- $\mu_{i}$ is the non-HIV related mortality rate among non-PWID
- $\bar{\mu}_{i}$ is the non-HIV related mortality rate among PWID (including drug-related mortality).

## Determining the number of partners for heterosexual transmission

Heterosexual interactions between main and casual partners can occur between any of the groups below and also with the general population. Any two arrowheads that are linked show an interaction if they are between a male/female subgroup.

**Supplementary Figure S3:** Schematic showing the different non-commercial heterosexual partnerships that can take place. Females from any compartment can have heterosexual main and casual partnerships with males from any group.

We model heterosexual transmission through main and casual partners among all 10 of the different subgroups. We assume that mixing is random between the different sub-groups, but that only a proportion of the main and casual partnerships are formed within the model and the rest are formed with the general population. We define this proportion for sub-group $i$ as $H_{i}^{p}$ ($p$ = partnership type, *p*=M, main or *p*=C, causal). However, as HIV prevalence in the general population is low we do not include this in the model.

Let $t_{i}^{p}$ be the proportion of partnerships of type $p$ captured by the model among females in group $i$ heterosexual mixing of a female in population sub-group $i$ and partnership type *p* and  $p$ be the proportion of parternships of type p captured by the model among males in group $i$. Then the mixing is defined for females by

$$t_{i}^{p}=\frac{H_{i}^{p}n_{i}^{p}N_{i}}{\sum_{k=1,2,3} H_{i}^{p}n_{k}^{p}N_{k}} if i=1,2,3$$

and for males by

$$\bar{t}_{i}^{p}=\frac{H_{i}^{p}n_{i}^{p}N_{i}}{\sum_{k=4,5,\ldots,10} {H_{k}^{p}n}_{k}^{p}N_{k}} if i=4,5,\ldots,10$$

where

- $n_{i}^{p}$ is the frequency of partners of type *p* of an individual in group $i$*.*
- $N_{i}$ is the total number of individuals in group $i$*.*

This gives the proportion of partnerships of type *p* among females/males an individual in sub-group $i$ has that are within the model.

It is necessary to balance the number of partnerships of type *p* between women and men within the model. The total number of females in sub-group $i$ with partnerships within the model is given by

$$H_{i}^{p}n_{i}^{p}N_{i}$$

with all parameters previously defined. Therefore, the total number of females with main partners within the model is given by

$$\sum_{k=1,2,3} H_{k}^{p}n_{k}^{p}N_{k}$$

This means the total number of partners that females have within the model with males in group $i$ is given by

$$\bar{t}_{i}^{p}\sum_{k=1,2,3} H_{k}^{p}n_{k}^{p}N_{k} for i=4,5,\ldots,10$$

In order to balance so that the same number of males have partnerships in the model, we assume that the number of partnerships males have within the model is dependent on the above expression. The above gives the total number of partners of type *p* that females in group $i$ have – therefore dividing by the total population size of group $i$ will determine the adjusted number of partnerships of type *p* that an individual in subgroup $i$ will have within the model

$$\bar{Hn}_{i}^{p}=\frac{t_{i}^{p}}{N_{i}} \sum_{k=1,2,3} H_{k}^{p}n_{k}^{p}N_{k} for i=4,5,\ldots,10$$

This is adjusted both in terms that it will (a) differ from the number of sexual partners of type $p$ that an individual in sub-group $i$ may have reported in the data as we are balancing the number of female and male partnerships and (b) is weighted such that it accounts for only the proportion of partnerships that females have with males within the model.

We therefore have that the adjusted proportion of males in group $i$ that have partnerships of type *p* with females in group $j$ within the model is

$$\frac{t_{i}^{p}}{N_{i}}t_{j}^{p}\sum_{k=1,2,3} H_{k}^{p}n_{k}^{p}N_{k}$$

which simplifies to

$$\frac{t_{i}^{p}}{N_{i}}H_{j}^{p}n_{j}^{p}N_{j}.$$

## Model setting and main data

We parameterize our model to Tijuana, Mexico, and utilize behavioural and epidemiological data from numerous local studies (Supplementary Table I). To parameterise the PWID population, the bio-behavioural *El Cuete II* (*ECII*, cross-sectional survey; 2005), *El Cuete III^12^* (*ECIII*, longitudinal study; 2006-2010) and *El Cuete IV* (*ECIV*, longitudinal study; 2010-2015) studies were used, with individuals recruited via respondent driving sampling (*ECII* and *ECIII*) or convenience sampling (*ECIV*). To parameterise the FSW population, behavioural intervention studies *Mujer Segura^13^* (*MS*, longitudinal study; 2003-2008) and *Mujer mas Segura^14,15^* (*MMS*, longitudinal study among FSW-PWID; 2008-2013) were used alongside *Parejas^16^* (longitudinal study; 2009-2014) and *MAPA^17,18^* (longitudinal study; 2010-2015) studies. Sampling methods included recruitment through outreach workers and municipal and community health clinics (*MS*), targeted sampling (*MMS*), targeted and snowball sampling (*Parejas*) and time location sampling (*MAPA*). *Sexo Seguro^19^* (*SS*, cross-sectional study; 2008) and *Hombre Seguro^20^* (*HS*, longitudinal intervention study; 2010-2013) were used to parameterise the client population. Clients were recruited using outreach workers (*SS*) and time-location sampling (*HS*). MSM were parameterised using data from *Proyecto H^21^* (cross-sectional study 2012) where individuals were recruited using RDS.

Supplementary Table I: Studies used for parameterisation of the model.

| **Study name** | **Years** | **Sample size at enrolment** | **Study type and Sampling methodology** | **HIV prevalence (%) in cross-sectional survey or at enrolment for longitudinal surveys (95%CrI)** | **Reference(s)** |
| --- | --- | --- | --- | --- | --- |
| ***PWID*** | | | | | |
| *El Cuete II (ECII)* | 2005 | 222 | Cross sectional – Respondent driven sampling | 2.7% (0.6 – 4.9%) |  |
| *El Cuete III (ECIII)* | 2006 – 2010 | 1056 | Longitudinal cohort – Respondent driven sampling | 4.5% (3.2 – 5.7%) | ^12^ |
| *El Cuete IV (ECIV)* | 2010 – 2015 | 734 | Longitudinal cohort – Convenience sampling | 3.5% (2.2 – 4.6%) | ^22^ |
| ***FSW*** | | | | | |
| *Mujer Segura* | 2003 – 2008 | 474 | Longitudinal cohort – Recruitment through outreach workers and municipal and community health clinics | 7.6% (5.2 – 10.1%) | ^13^ |
| *Parejas* | 2009 – 2014 | 106 | Longitudinal cohort – Targeted and snowball sampling | 3.8% (0.1 – 7.5%) | ^16^ |
| *MAPA* | 2010 – 2015 | 301 | Longitudinal cohort – Time location sampling | 2.7% (0.8 – 4.5%) | ^17,18^ |
| ***FSW-PWID*** | | | | | |
| *Mujer mas Segura* | 2008 – 2013 | 284 | Longitudinal cohort – Targeted sampling | Eligibility criteria: all HIV -ve at baseline | ^14,15^ |
| ***Clients of FSW*** | | | | | |
| *Sexo Seguro* | 2008 | 211 | Cross sectional – Recruitment using outreach workers | 5.7% (2.6 – 8.9%) | ^19^ |
| *Hombre Seguro* | 2010 – 2013 | 203 | Longitudinal cohort – Time location sampling | Eligibility criteria: all HIV -ve at baseline | ^20^ |
| ***MSM*** | | | | | |
| *Proyecto H* | 2012 | 191 | Cross sectional – Respondent driven sampling | 17.3% (11.9 – 22.7%) | ^21^ |

Abbreviations: PWID: people who inject drugs (have injected in the past 6 months); FSW: female sex workers; MSM: men who have sex with men.

## Model parameterisation

Injecting behaviour parameters are based on data from *ECIV*. The number of injections per year among male and female PWID was assumed stable at 1,440 (IQR: 1,080-1,440). We assume a heightened number of injections per month (RR: 1.3 (95%CI: 1.0-1.66)) for MSM who inject drugs, but fewer (0.87 (95%CI: 0.79-0.97)) for female PWID that are not a FSW. Overall, 67.9% (63.3-72.5%) of injectors are assumed to have receptively shared syringes in the past 6 months, with greater sharing among clients (RR: 1.4 (1.3–1.6)), MSM (RR 1.3 (1.1-1.6)) or FSW (RR: 1.1 (1.0 – 1.2)) that inject drugs. Out of those who have receptively shared needles, for all groups we assume that 45.9% (95%CI: 38.7-53.2%) have done so at last injection.

Sexual behaviour parameters are based on data from all surveys previously mentioned, with data sources given in Table I of the main paper and Supplementary Table II. We assume 62.3% (95%CI: 55.4-69.3%) of MSM have at least one main male partner, with them having a median of 2 (IQR: 1-2) main partners. We assume that 60% (95%CI: 53–67%)) of MSM have at least one casual partner, with a median of 12 (IQR:0-48) casual partners per year. For these partnerships, we assumed 25.2 (IQR: 10.2–78) sex acts in the past year with main partners and 1.1 (IQR: 1-2) sex acts with each casual partner in the past year. Baseline consistency of condom use among MSM was 60.8% (95%CI:52.3-69.2%) and 77.9% (95%CI:71.1-84.8%) for main and casual partners, respectively. We assume that condom use increased from 1987, with baseline consistency of condom use of 60.8% (95%CI: 52.3-69.2%) or 77.9% (95%CI: 71.1-84.8%) with main and casual partners, respectively.

We assume that each key population have a given percentage of heterosexual main or casual partners within the model. We assume that a given percentage of these partners are PWID or clients, and the average number of main or casual partners each group has for vaginal and anal sex (Main paper – Table I). Note that these differ by key population sub-group. We assume that condom use increased from 1987 between main and casual partners; condom use is increased to the average baseline condom use across all groups for vaginal and anal sex between casual partners and is assumed to be between 23.3-60.3% and 5.2-88.7% based on survey data, respectively with lower condom use assumed between main partners (8.7-28.3% and 4-32.3% for vaginal and anal sex, respectively).

We assumed that 84.5% (95%CrI: 81.7 – 87.4%) of transactions from sex work between

commercial sex acts between clients non-PWID and FSW are vaginal, with this decreased by 0.91 (95%CrI: 0.83 – 0.99) for client PWID. Condom use for transactional sex between FSW and clients is assumed to have increased from 1987 up to an average of the reported condom use between FSW and clients. We assume FSW used condoms 48.3 – 83.8% of the time (regardless of type of sex act) and clients used condoms 54.8% (48.3 – 31.3%) and 45.8% (32.7 – 58.9%) of the time for transactional vaginal and anal sex acts, respectively. We take the average of the sampled values to ensure that FSW and clients have the same percentage of condom use in sexual contacts.

We assume that ART started in Tijuana in 2003 and has since scaled-up among different key populations, with the model calibrating a recruitment rate onto ART required to achieve 2-18% ART coverage amongst PWID and FSW in 2012 and 30% (15-45%) coverage amongst MSM and clients in 2017, remaining constant thereafter. We assume that ART slows disease progression by 70-80%^7-10^ and assume a loss to follow-up between 0.67-13.35/100pyrs^11^.

Upon entry to the model. we assume a life expectancy of individuals in the model based on those aged 15, using data from the UN World Population Prospects database over 2015-2020. The modelled population does not have an upper age limit, with FSWs, PWID and clients leaving the model when they cease these risk behaviours. The average duration that they remain in the risk groups is given in Supplementary Table II and is based on survey data. MSM remain in the modelled population for life.

All parameters used and their uncertainty distributions (95% confidence interval or interquartile range) are given in Table I of the Main paper and Supplementary Table II.

**Supplementary Table II:** Additional parameters for the model which are not given in the main paper. Prior distributions and their ranges are given for each parameter.

| Parameter | Prior distribution | Distribution | Data information |
| --- | --- | --- | --- |
| General population demographic information | | | |
| Total population size | 1,161,521 – 1,742,281 | Uniform | Tijuana pop size: 1.987 million. In Mexico 26.93% aged < 15. Use point estimates and then +/- 20%  Used to calibrate the rate that individuals enter the model. |
| Proportion of population female | 41.4 – 62.1% | Uniform | 51.74% of population female. +/- 20% |
| General population prevalence | 0.05% (95% CI: 0.01 – 0.1) | Truncated normal | ^1^ |
| Demographic information | | | |
| Mortality rate (non-PWID) | 0.0157 (0.0125 – 0.0188) | Truncated normal | Life expectancy at 15: 64.23 from 2015-2020.  0.0157 +/-20% |
| Mortality rate (PWID) | 0.0394 (0.0327 – 0.0460) | Truncated normal | ECIV |
| HIV transmission parameters | | | |
| Transmission hazard per 100 pyrs in acute phase of infection | 276 (131 – 509) | Log-normal | ^5^ |
| Transmission hazard per 100 pyrs in latent phase of infection | 10.6 (7.6 – 13.3) | Log normal |  |
| Transmission hazard per 100 pyrs in pre-AIDS phase of infection | 76 (41.3 – 128) | Log-normal |  |
| Duration in acute phase of infection | 2.9 months (1.2 – 6.0) | Log-normal |  |
| Duration in pre-AIDS phase of infection | 9.0 months (4.8 – 14.0) | Log-normal |  |
| Duration in AIDS phase of infections | 10.0 months (7.0 – 12.7) | Log normal |  |
| Duration from seroconversion to AIDS (to determine duration in latent phase of infection) | 9.4 years (IQR: 5.5 – 10.1) | Triangular | ^6^ |
| Injecting transmission rate | 0.0001 – 0.0092 | Uniform | ^2^Extended range. |
| Risk of HIV infection per receptive vaginal sex act with an infected partner | 0.00081 (0.00060 – 0.00109) | Log-normal | ^3^ |
| Increase in receptive transmission for anal sex acts | 2 - 18 | Uniform | ^4^ |
| Risk of HIV infection per insertive vaginal sex act with an infected partner | 0.00042 (0.00013 – 0.00141) | Log-normal | ^3^ |
| Increase in insertive transmission for anal sex acts | 1 - 2 | Uniform | ^4^ |
| Condom efficacy | 70 – 95% | Uniform |  |
| ART parameters | | | |
| Reduced progression rate if on ART | 70-80% | Uniform | ^7-10^ |
| Loss to follow-up rate from ART | 0.67 – 13.35/100pyrs | Uniform | ^11^ |
| Decrease in sexual transmission if on ART | 85 – 95% | Uniform |  |
| Decrease in injecting transmission if on ART | 25 – 75% | Uniform |  |
| Uninformative priors:  Rate initiate ART if FSW  Rate initiate ART if PWID  Rate initiate ART if MSM  Rate initiate ART if client | 0 – 0.3  0 – 0.3  0 – 0.3  0 – 0.3 | Uniform  Uniform  Uniform  Uniform | Note – to calibrate to:  2-18% among FSW  2-18% among PWID  15-45% among MSM  15-45% among clients |
| Date of ART initiation | 2003 | N/A |  |
| Additional Commercial sex parameters – Female sex workers and their clients | | | |
| Duration of sex work if FSW non-PWID | 2-24yrs | Uniform | MAPA – extended IQR |
| Relative increased in duration of sex work if FSW PWID | 1.8 (1.5 – 2.2) | Truncated normal | MAPA |
| Duration of being a client if non-PWID | 8 – 54yrs | Uniform | Hombre Seguro – extended IQR |
| Duration of being a client if PWID | 16 – 62yrs | Uniform | Hombre Seguro – extended IQR |
| Additional injecting drug use parameters | | | |
| Duration of injecting among female PWID | 5 – 40yrs | Uniform | ECIV |
| Duration of injecting among male PWID | 12 – 50yrs | Uniform | ECIV |
| Additional parameters relating to heterosexual main partnerships | | | |
| Percentage of each key population with a main partner  Female PWID  FSW non-PWID  Male PWID, non-client, non-MSM,  Male PWID, non-client, MSM  Clients (all groups)  MSM (non-client, nonPWID) | 70.4% (63.6 – 77.1%)  56.1% (50.5 – 61.8%)  41.3% (35.1 – 47.6%)  Assume same as male PWID, non-client, non-MSMS  51.1% (43.7 – 58.5%)  17.7% (12.1 – 23.3%) | Truncated normal  Truncated normal  Truncated normal  Truncated normal  Truncated normal | No significant difference between the proportion of PWID non-FSW and PWID FSW that have a main partner – use overall data for female PWID  MAPA  ECIV  Hombre Seguro  Proyecto H |
| Percentage of female key population with a main partner whose main partner is also an injector  Female PWID non-FSW  Female PWID FSW  FSW non-PWID | 7.08% (2.3 – 11.9%)  64.1% (48.3 – 79.9%)  3.2% (0 – 6.9%) | Truncated normal  Truncated normal  Truncated normal | ECIV  MAPA  MAPA |
| Percentage of FSW non-PWID with a main partner whose main partner is a client | 2.8% (0 – 6.0%) | Truncated normal | Parejas |
| Additional parameters relating to heterosexual casual partnerships | | | |
| Percentage of each key population with a casual partner  Female PWID non-FSW  Female PWID FSW  FSW non-PWID  Male PWID, non-client, non-MSM  Male PWID clients (MSM/non-MSM)  Clients (non PWID, non-MSM)  MSM (non-client, nonPWID) | 18.1% (6.0 – 26.5%)  OR: 5.8 (2.9 – 11.6)  Assume same as PWID FSW  26.9% (21.2 – 32.5%)  55.0% (41.6 – 67.9%)  33.1% (25.7 – 42.1%)  15.7% (10.5 – 20.9%) | Truncated normal  Log normal  Truncated normal  Truncated normal  Truncated normal  Truncated normal | ECIV  ECIV  ECIV  Sexo Seguro  Sexo Seguro  Proyecto H |
| Percentage of female key population with a casual partner whose casual partner is also an injector  Female PWID non-FSW  Female PWID FSW  FSW non-PWID | 18.1% (6.0 – 26.5%)  42.6% (29.9 – 55.4%)  Assume same as PWID non-FSW | Truncated normal  Truncated normal | ECIV  ECIV |
| Percentage of FSW non-PWID with a casual partner whose casual partner is a client | Assume same as for main partner |  |  |

Priors for the number of individuals in each overlapping group were estimated. We firstly assumed a total of 10,000 PWID and used survey data to estimate each of the different population sizes. The different stages of this are shown in Supplementary Table III. For each sampled parameter set the population size of each of the key populations was estimated.

**Supplementary Table III:** Method for estimating the size of each key population and their overlaps using the different survey data. In the example below the mean or mid-point of the estimate is used.

| Step | Methods | Example |
| --- | --- | --- |
| 1 | - Start with 10,000 PWID - 15.0% (95%CI: 12.8-17.1%) of PWID are female (El Cuete II) - Results in the total number of female and male PWID | 10,000*0.15  1,500 female PWID/8,500 male PWID |
| 2 | - 38.6% (30-8.- 46.4%) of female PWID are female sex workers (El Cuete III) - Results in the overlap of FSW and PWID | 1,500*0.386  579 PWID-FSW |
| 3 | - 26.6% (26.1 – 31.6%) of FSW have injected in the past 6 months - Results in the total number of FSW | 579/0.266  2,177 FSW |
| 4 | - Calculate the number of FSW who are not PWID - Results in the number of FSW non-PWID | 2,177 – 579  1,598 FSW non-PWID |
| 5 | - Balance the number of clients seen by FSW and number of FSW seen by clients to get the total number of clients based on the population size of FSW i.e.   *Number of FSW*Number of clients seen = Number of clients*Number of FSW seen*   - FSW PWID see 180 (IQR: 60–360) clients every year (MMS) - FSW non-PWID see 120 (IQR: 60–240) clients every year (MMS) - Clients see 12 (IQR: 6 – 24) FSW each year (Sexo Seguro) - Results in the total number of clients | Clients seen by FSW-PWID  (579*180) = 102,420  Clients seen by FSW non-PWID  (1,598*120) = 191,760  (102,420 + 191,760)/12  24,515 clients |
| 6 | - 8.3% (5.8 – 10.9%) of male PWID are clients (El Cuete IV) - Results in the total number of PWID-clients (both MSM and non-MSM) | 8,500*0.083  706 PWID-clients |
| 7 | - 0 – 47.4% of clients that are PWID are MSM (range over PWID and client studies) - Results in the number of PWID-client-MSM and PWID-client-non-MSM. | 706*0.237 (mid point of range)  167 PWID-client-MSM  706 – 167  539 PWID-client-nonMSM |
| 8 | - 7.2% of male PWID have sex with men (El Cuete IV) - Results in the total number of PWID-MSM. Can use previous totals to calculate PWID-nonClient-MSM | 8,500*0.072  612 PWID MSM  612 – 167   1. PWID-nonClient-MSM |
| 9 | - 5.6% (2.2 – 9.0%) of clients have sex with men (Hombre Seguro). Use the number of clients to calculate the number of client-nonPWID MSM - Results in the number of client non-PWID MSM | 24,515*0.056 - 167  1205 client non-PWID MSM |
| 10 | - Use the previously calculated population sizes to determine the number of clients in no other risk group - Results in to total number of client non-PWID non-MSM | 24,515 – 167 – 539 – 1,205  22,604 client non-PWID nonMSM |
| 11 | - 12.6% (7.8 – 17.2%) of MSM are PWID (Proyecto H) - Results in the total number of MSM | (434 + 167)/0.126  4,770 MSM |
| 12 | - Use the previously calculated population sizes to determine the number of MSM in no other risk group - Results in the total number of non-client non-PWID MSM | 4,770 – 1,205 – 434 – 167  2,964 non-client non-PWID MSM |

**Figure S4:** Model projected overlap of key populations in Tijuana. The median estimated total population sizes and their overlaps in 2020 are shown, with the size of ellipse proportional to the population size of the group (i.e. there are in total 1225 female PWID, 1713 FSW, and 476 females who are both PWID and FSW). The figure also shows the percentage of the total key population size in brackets for each of the key population sizes and their overlaps.


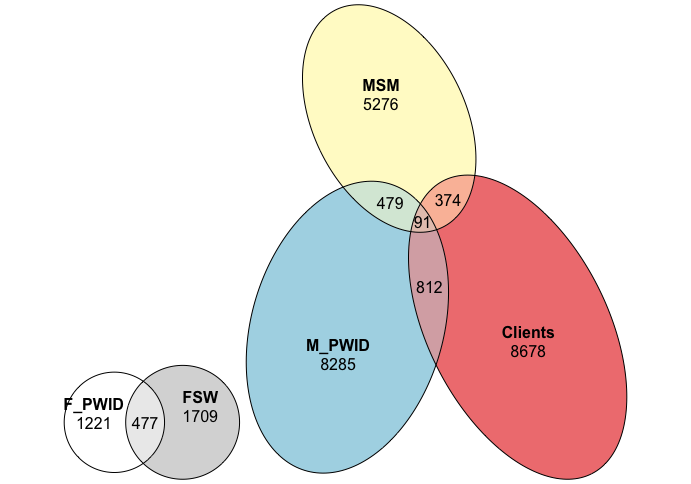


(30.2%)

(31.7%)

(19.3%)

(1.7%)

(1.4%)

(3.0%)

(0.3%)

(6.2%)

(4.5%)

(1.7%)

## Model calibration

The model is calibrated to the HIV prevalence among PWID, FSW and MSM (main paper Table I), and ART coverage among all key populations (2-18% among PWID and FSW in 2012, and 15-45% among MSM and clients in 2017). An Approximate Bayesian computation sequential Monte Carlo (ABC SMC) scheme was used to obtain 5,000 full parameter sets that fit the data. A random sample of 5,000 parameter sets was obtained from their prior distributions. At subsequent iterations, parameter sets from the previous iteration were sampled with replacement and perturbed so as to be within the prior ranges initially given. This process was repeated until the sum of the log-likelihood of the model was less than our desired criteria, which was determined by finding the log-likelihood of values equivalent to the maximum and minimum of the 95% confidence intervals of the data being calibrated to and taking the smallest of these.

At each iteration of the ABC, uninformed parameters were calculated analytically using the population size estimates and behavioural data. Any parameter set which could not produce biologically realistic rates (i.e. had rates of movement less than 0 to obtain the population size estimates) were discarded.

## Log-likelihood calculation

To calculate the log-likelihood of the data the following expression is use:

$$L\left( x | a,b \right)=-\sum_{i} \log\left( \frac{1}{B\left( a_{i},b_{i} \right)}x_{i}^{a-1}\left( 1-b_{i} \right)^{b_{i}-1}(x_{i}) \right)$$

where $B(\cdot,\cdot)$ is the beta function, $a$ and $b$ are vectors which are formed of the data giving the number of HIV sero-positive and sero-negative individuals from the studies at different time points and $x$ is the corresponding vector giving the model estimate for prevalence in the given population at a point in time.

### OST and NSP coverage in Tijuana

While needle and syringe programmes (NSP) have been in operation in Tijuana since 2004, receipt of high coverage NSP (one or more sterile syringes for each injection) is very low and irregular. Nationally, only an estimated 4 syringes are distributed per PWID per year, apart from a brief period in 2011-2013 when the Global Fund support of NSP increased provision to an estimated 20 syringes per PWID annually^23^. Similarly, opioid agonist therapy (OAT) coverage is very low among PWID (<10% receiving methadone across 2006-2010).

# Results

## Model calibration – ART coverage

The model was calibrated to ART coverage amongst PWID, FSW (2-18% coverage for both groups in 2012), clients and MSM (15-45% coverage for both groups in 2017). Supplementary Figure S5 shows the model projections and data for ART coverage amongst the four key populations that the model was calibrated to.

**Supplementary Figure S5:** ART coverage projections and available data estimates for (a) PWID; (b) FSW; (c) MSM and (d) clients in Tijuana, Mexico. The model was calibrated to HIV prevalence data amongst PWID, FSW and MSM and ART coverage data for all four key populations (denoted by vertical lines showing the range). ART coverage was assumed to start in 2003. Pale grey lines show the model projections for each of the runs, solid black lines show the median of model run, dashed black lines show the interquartile range of model runs, and dot-dashed lines show the 95% credibility intervals. Note survey acronyms used in figures: ECIII – El Cuete III; EC IV – El Cuete IV; MS – Mujer Segura; MAPA – Salud de MAPA. Note different y-axis.

(a) (b)


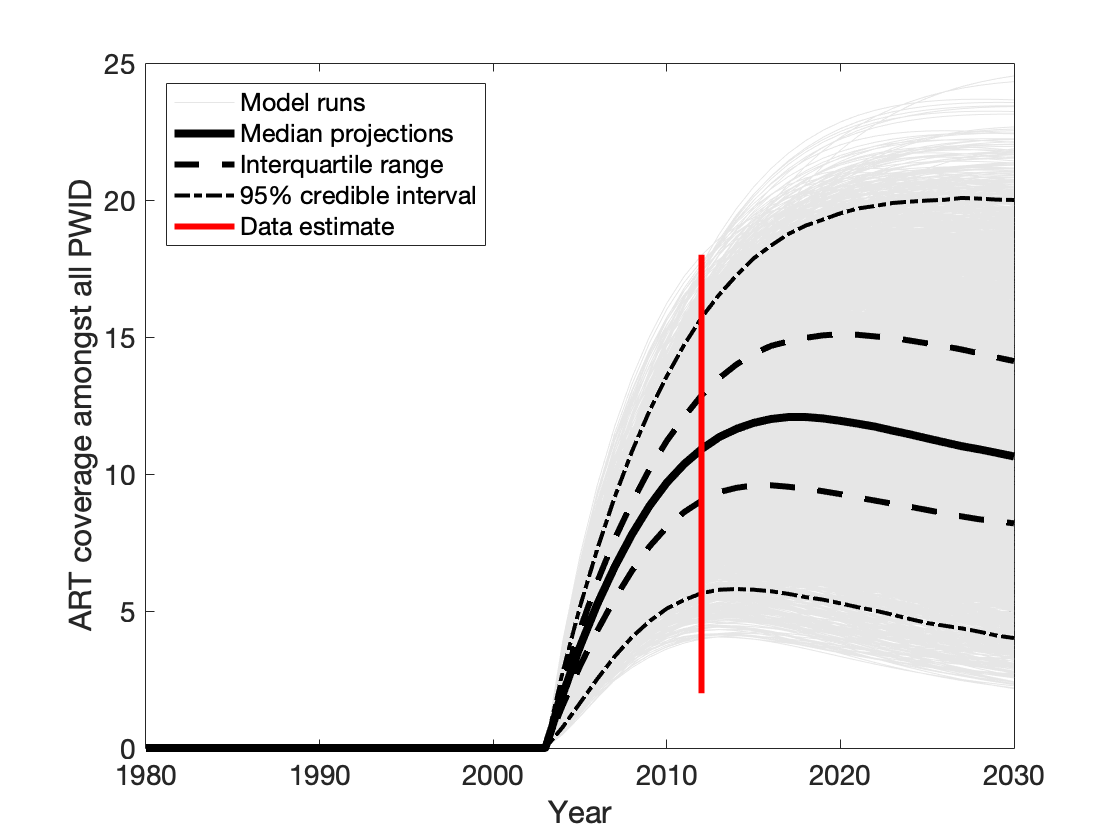

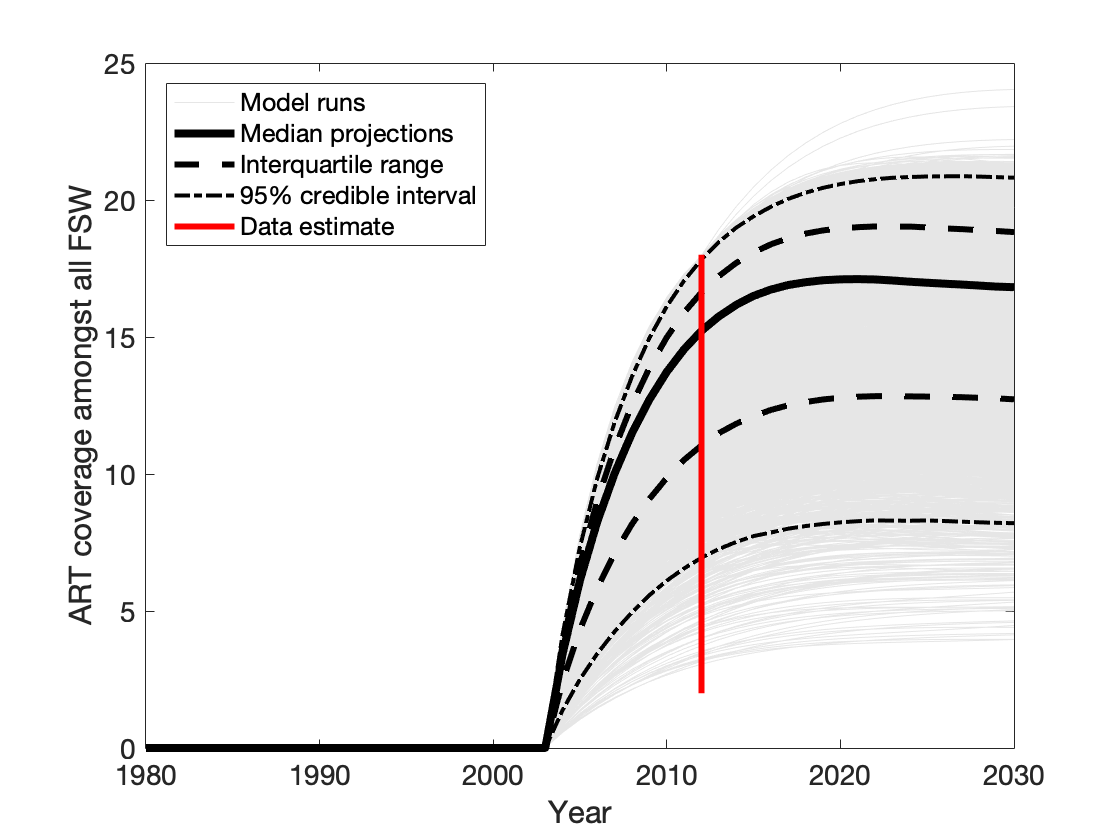


(c) (d)


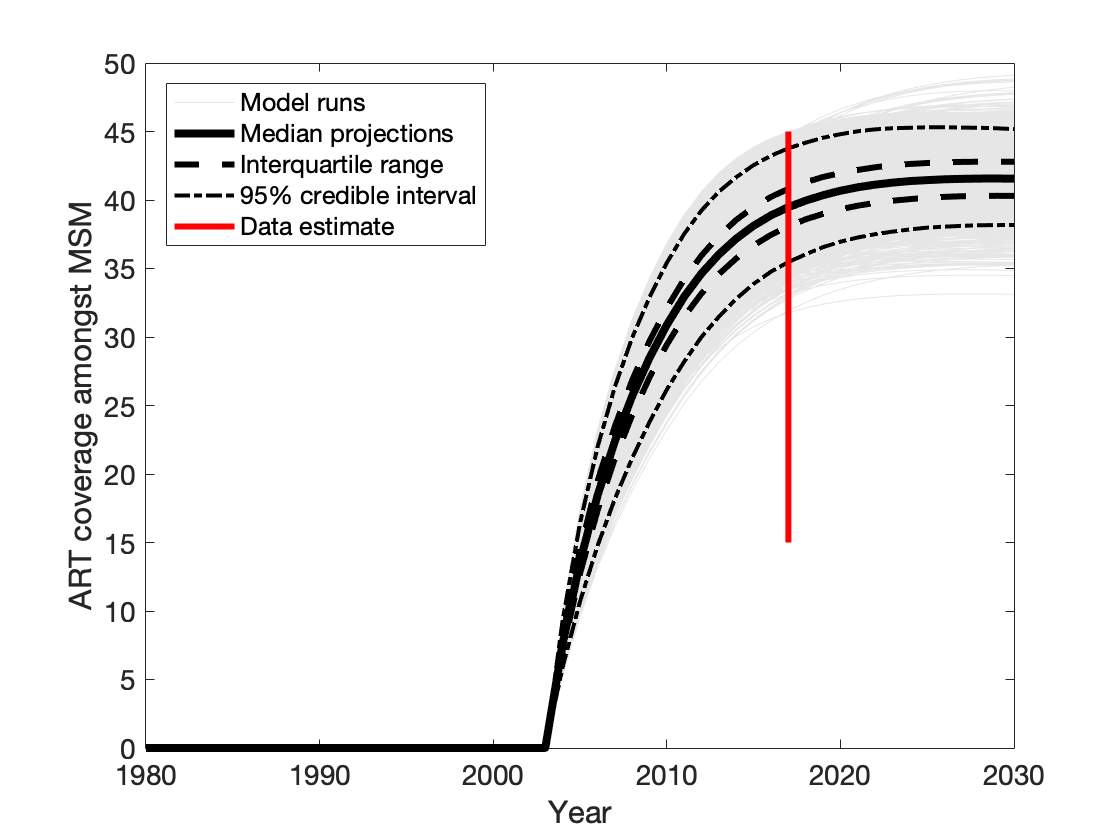

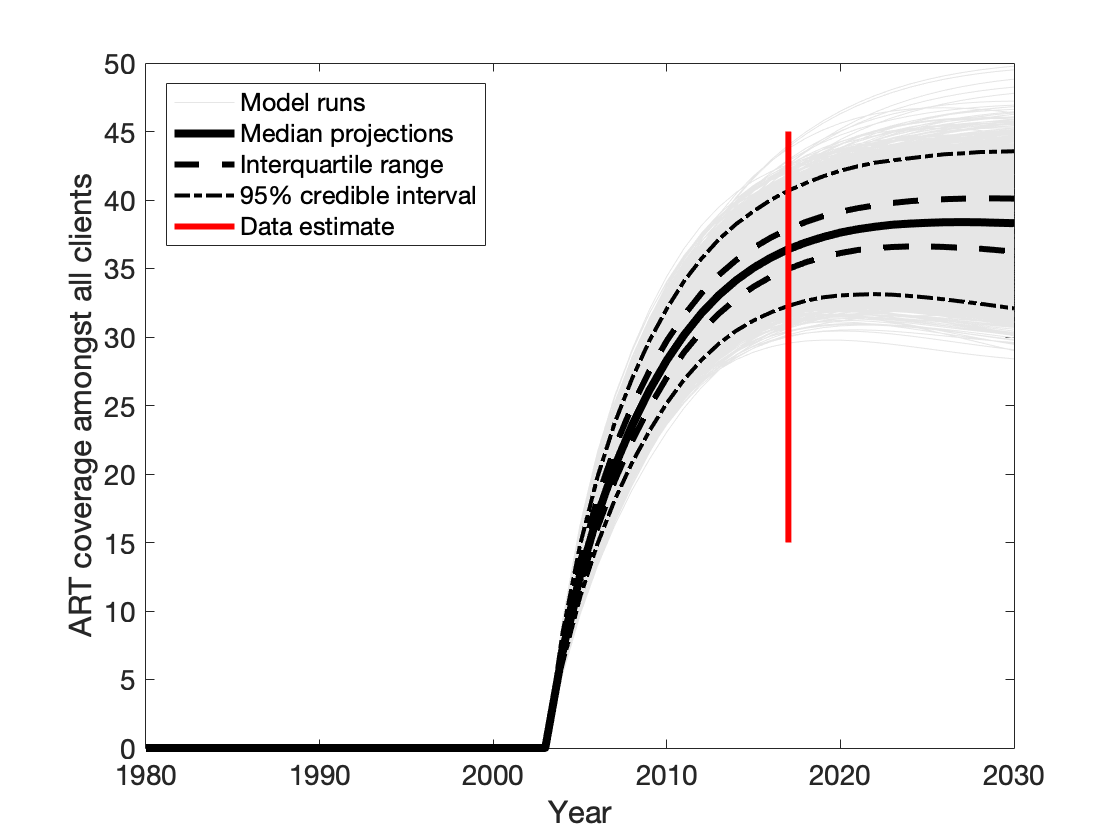


## Model validation

The data that is currently used for model validation is HIV incidence among PWID, FSW and clients, and HIV prevalence among PWID by gender and FSW by injecting status.

We decided to calibrate the model to overall prevalence in the different key populations rather than incidence as (a) there were more prevalence data points to calibrate the model to, and (b) the prevalence estimates were deemed to be more robust. Therefore, we chose to use incidence data solely for model validation.

For the prevalence validation data (HIV prevalence among PWID by gender and among FSW by injecting status), there is a similar level of uncertainty in the stratified data as in the overall data, and so using this data is unlikely to reduce the uncertainty in the model projections. Indeed, additional analyses have shown that included these stratified prevalence estimates does not unduly affect the HIV prevalence in 2020 that the model projects or the uncertainty around those prevalence projections.

### HIV incidence

Supplementary Figure S6 shows HIV incidence among PWID, FSW and clients. Whilst not calibrated to incidence among any key population, the incidence data validates the model fits. Note that there is no incidence estimate among MSM in Tijuana.

**Supplementary Figure S6:** HIV incidence projections and available data estimates for (a) PWID; (b) FSW; (c) MSM and (d) clients in Tijuana, Mexico. The data for each key population is denoted by circles (mean) and vertical lines (95% confidence intervals), although the model was not calibrated to this data. Pale grey lines show the model projections for each of the runs, solid black lines show the median of model run, dashed black lines show the interquartile range of model runs, and dot-dashed lines show the 95% credibility intervals. Note survey acronyms used in figures: ECIII – El Cuete III; EC IV – El Cuete IV; MS – Mujer Segura; MAPA – Salud de MAPA.

(a) (b)


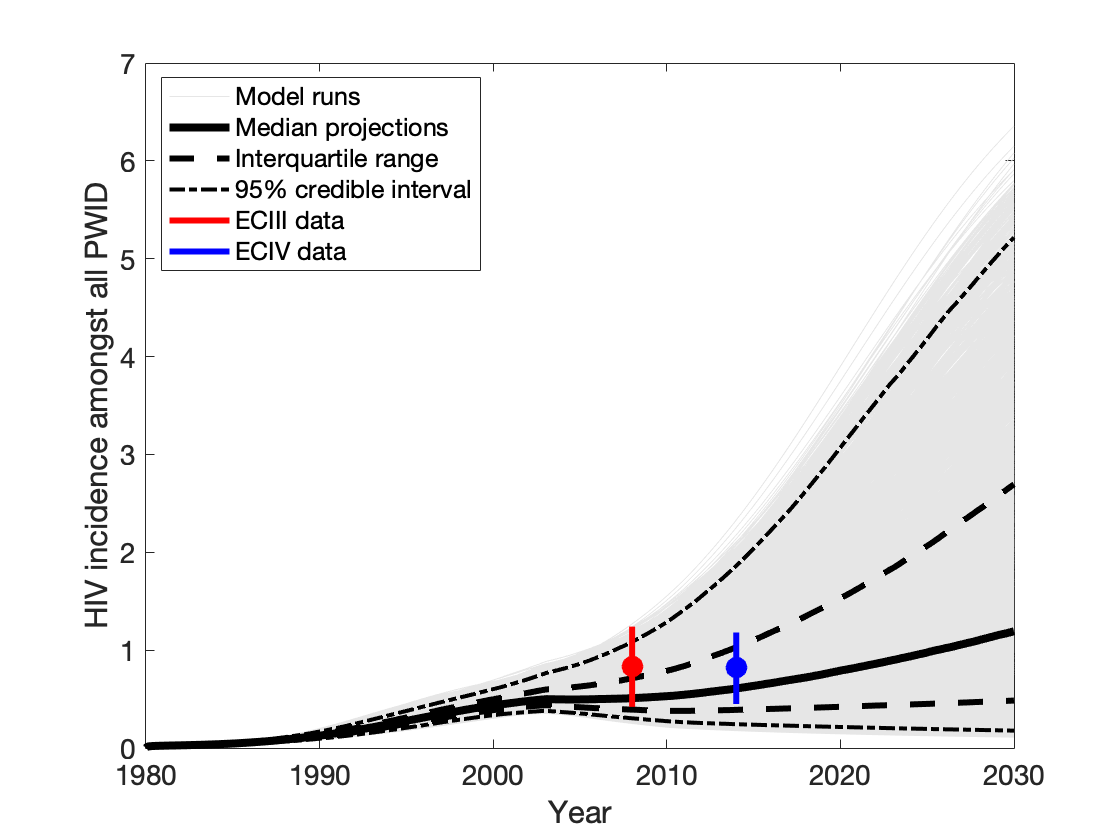

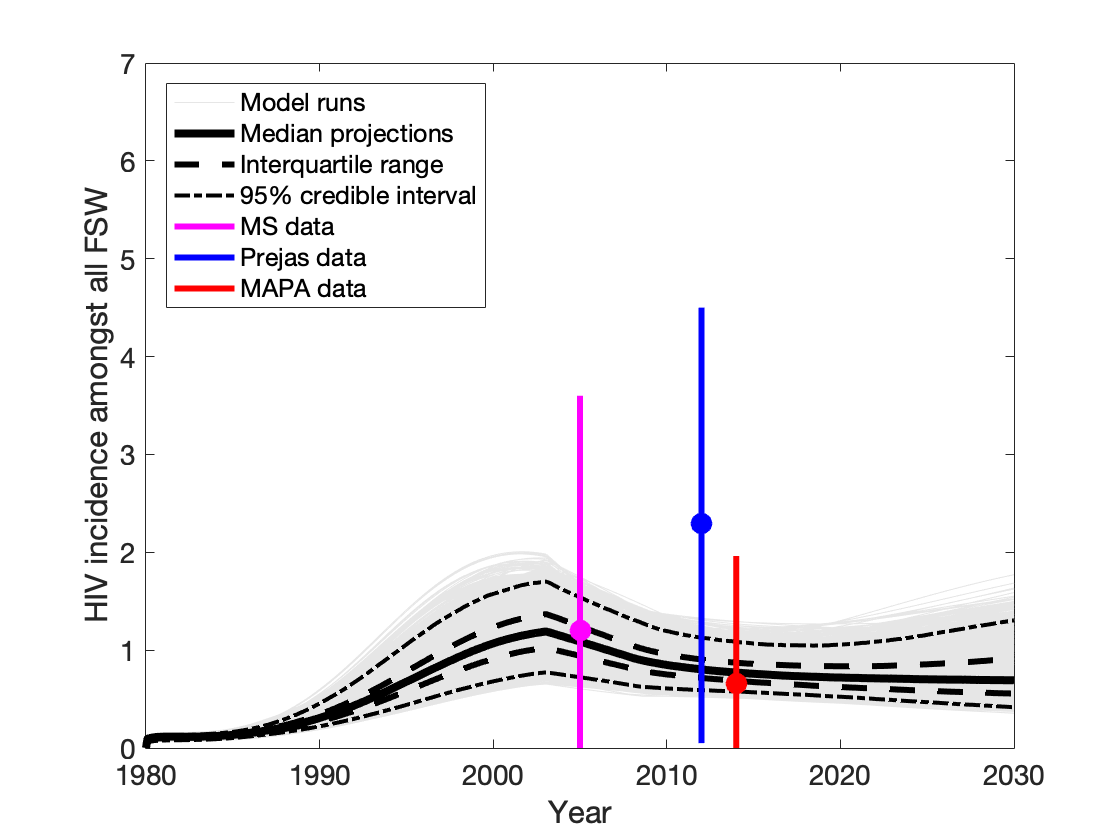


(c) (d)


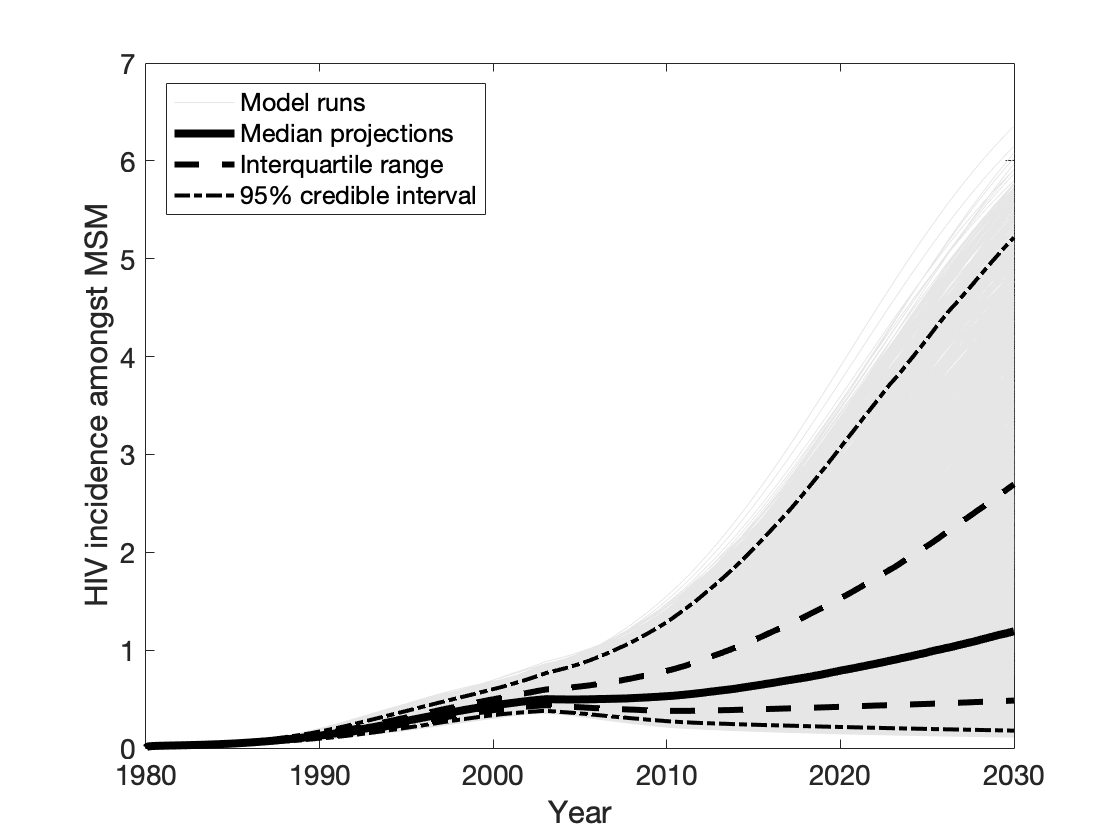

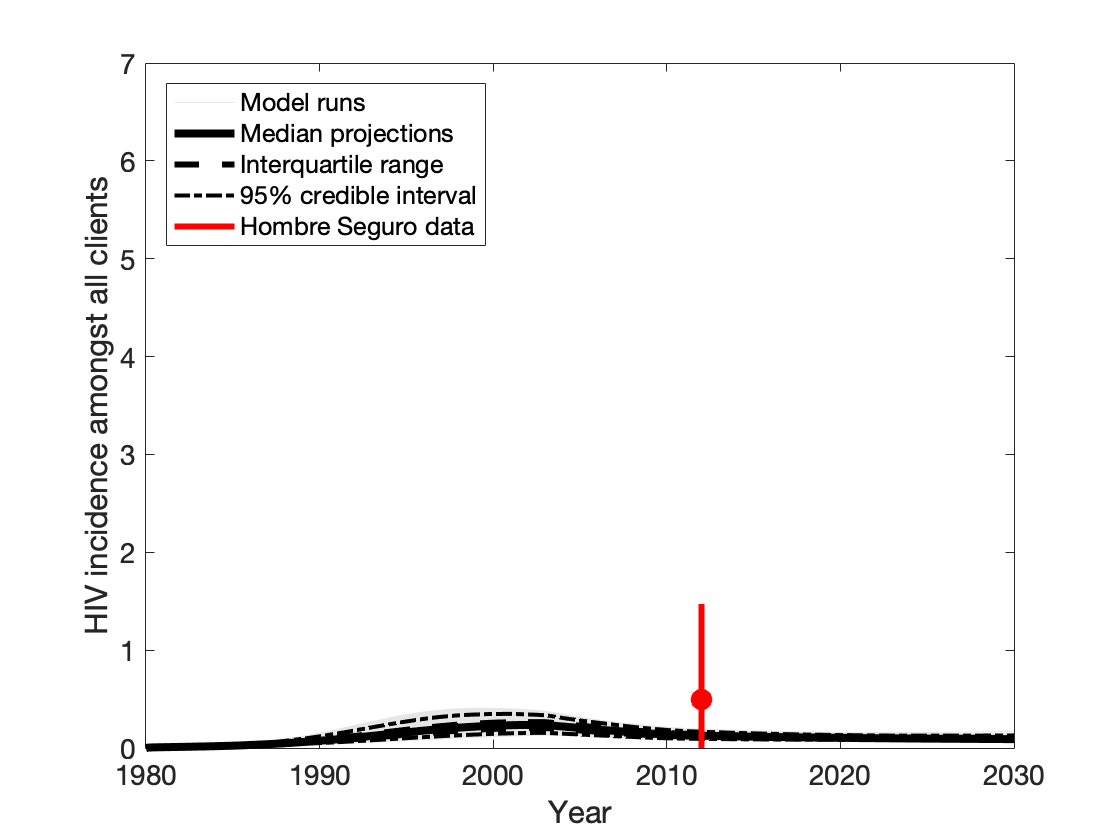


### HIV prevalence by groups

Supplementary Figure S7 shows HIV prevalence among PWID by gender, and HIV prevalence among FSW by PWID status. Although calibrated to overall prevalence among PWID and FSW, the model can replicate the HIV epidemic among female PWID, male PWID and FSW when stratified by injecting status (Supplementary Figure S5).

**Supplementary Figure S7:** HIV prevalence projections and available data estimates for (a) male PWID; (b) female PWID; (c) FSW PWID and (d) FSW non-PWID in Tijuana, Mexico. The data for each key population is denoted by circles (mean) and vertical lines (95% confidence intervals), although the model was not calibrated to this data. Pale grey lines show the model projections for each of the runs, solid black lines show the median of model run, dashed black lines show the interquartile range of model runs, and dot-dashed lines show the 95% credibility intervals. Note survey acronyms used in figures: ECII – El Cuete II; ECIII – El Cuete III; EC IV – El Cuete IV; MS – Mujer Segura; MAPA – Salud de MAPA; MMS – Mujer Mas Segura.

(a) (b)


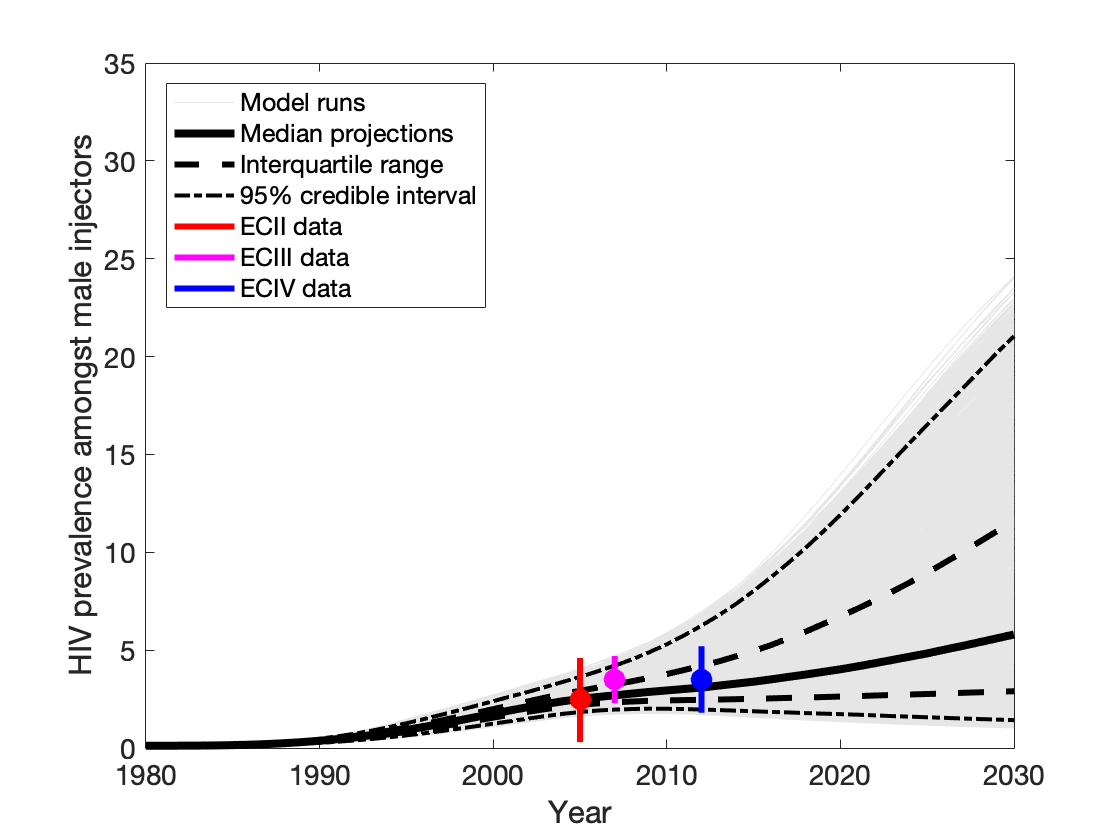

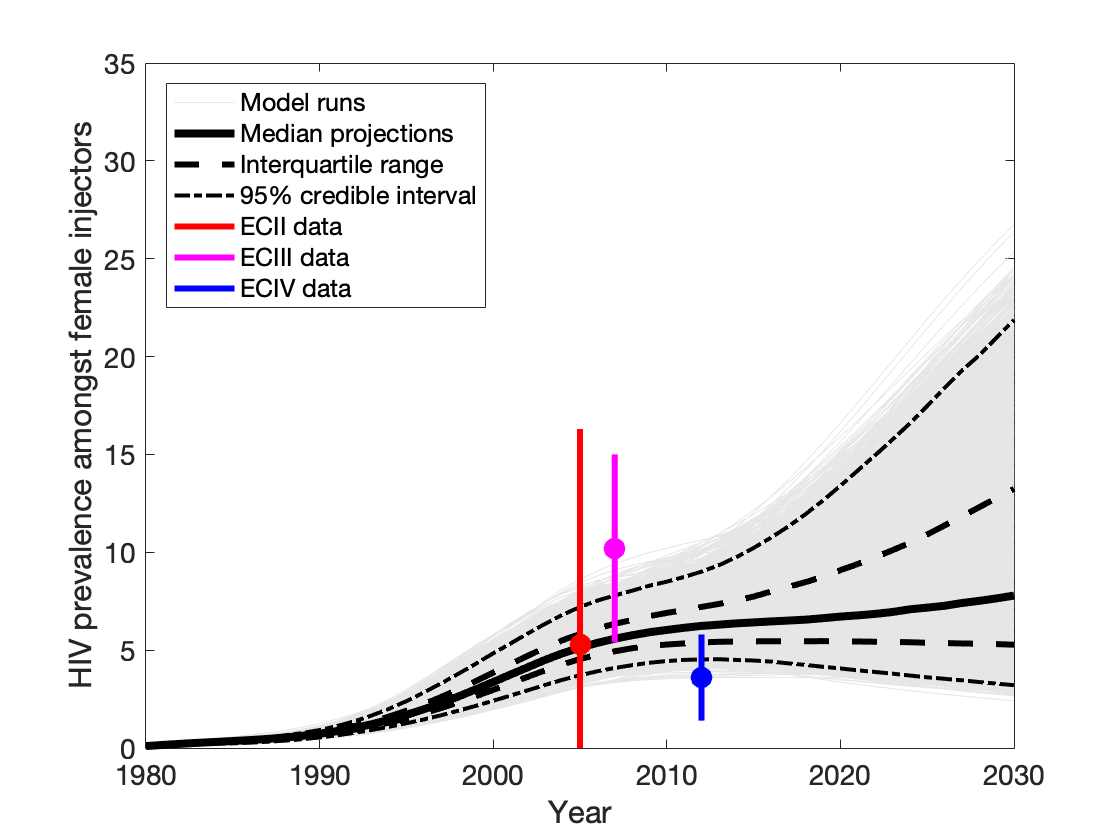


(c) (d)


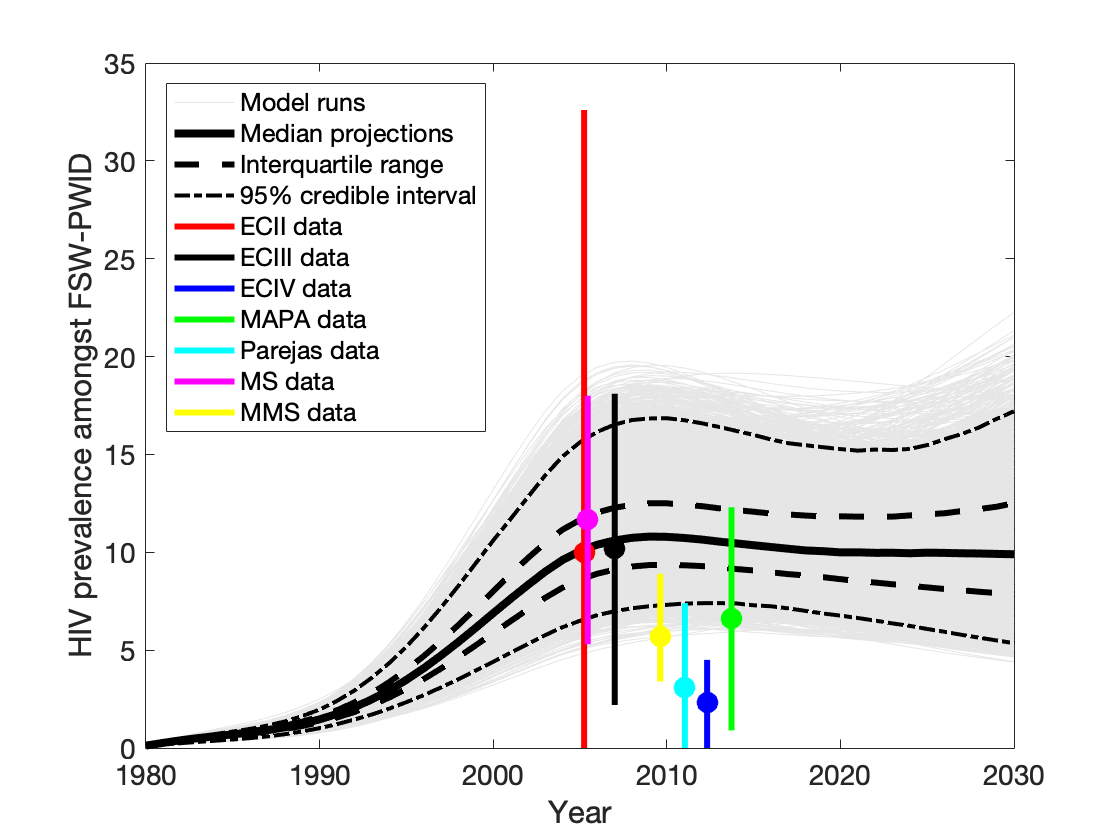

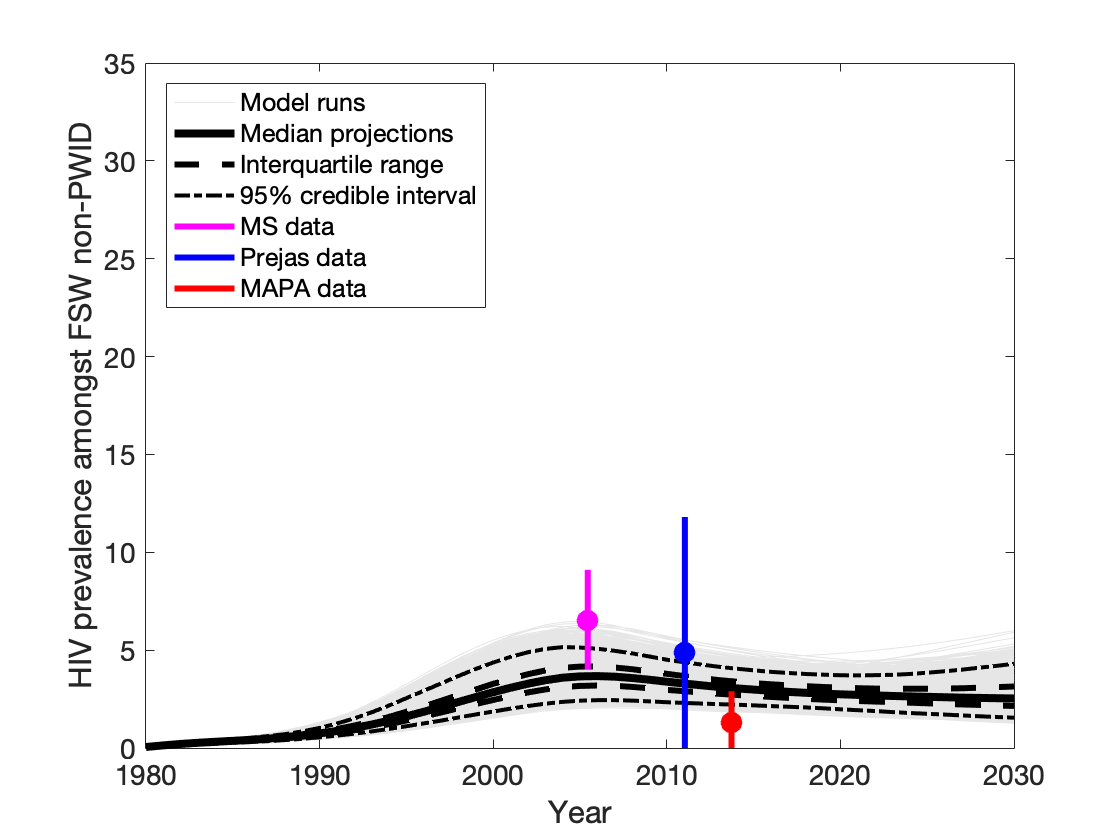


## Contribution of different risk behaviours to HIV transmission

Supplementary Figure S8 shows the inverse relationship that exists between the contribution of unsafe injecting drug use and the contribution of unprotected sex between men accounting for new HIV infections.

**Supplementary Figure S8:** (a) The contribution or population attributable fraction of unsafe injecting drug use and unprotected sex between men for the 5,000 different model runs. (b) The contribution or population attributable fraction of unsafe injecting drug use, unprotected sex between men and unprotected commercial sex as the HIV transmission probability increases for the 5,000 different model runs. Note: The population attributable fraction is estimated as the percentage decrease in new HIV infections among all key populations when HIV transmission risk due to specific risk behaviours is removed over 2020-2030.

(a) (b)


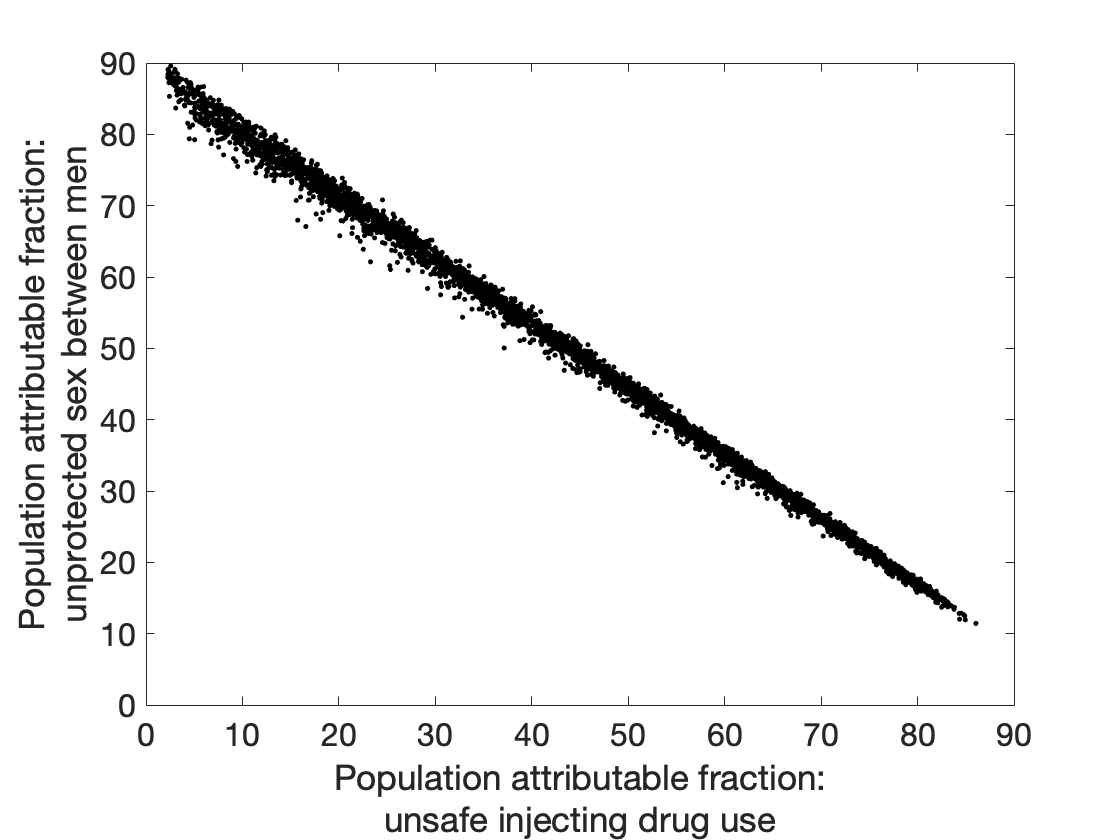

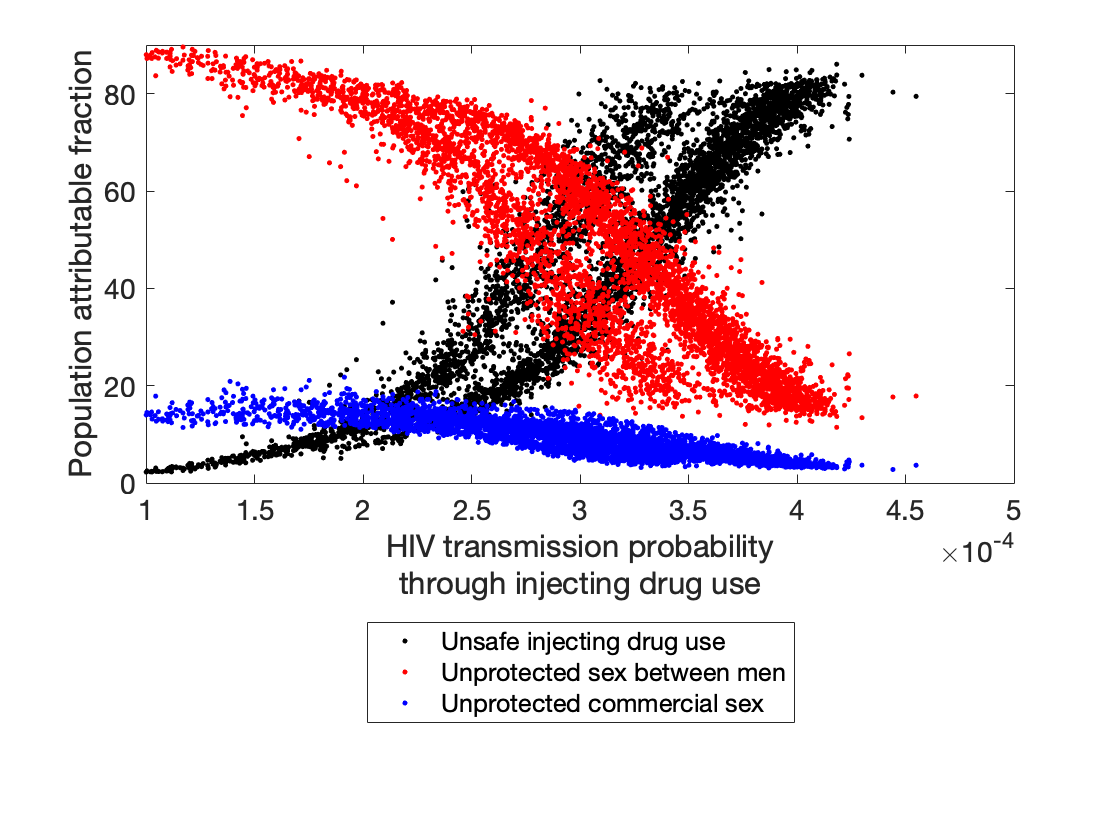


## Sensitivity analysis

Supplementary Figure S9 shows the population attributable fraction for the Baseline model runs (shown in the main paper) and two sensitivity analysis; firstly estimating the population attributable fraction of assuming that MSM-PWID only mix with each other when injecting and not other PWID, and secondly estimating the population attributable fraction if the ART coverage among MSM and clients were at national levels for men (70% coverage).

**Supplementary Figure S9:** The contribution of different risk behaviours to HIV transmission in Tijuana. This is estimated as the percentage decrease in new HIV infections among all key populations when HIV transmission risk due to specific risk behaviours is removed over 2020-2030 in Tijuana. Boxes show the 25^th^, 50^th^ and 75^th^ percentiles and the whiskers indicating the 2.5^th^-97.5^th^ percentiles over the 5,000 baseline model fits. The different risks removed are no injecting HIV transmission risk; no HIV transmission risk due to sex between men; no HIV transmission risk due to commercial sex; and no HIV transmission risk due to heterosexual main and casual partnerships. The scenarios are (1) assortative mixing among PWID, such that MSM who inject only inject with other MSM who inject (not other PWID) and (2) increased coverage of ART among MSM and clients (national levels; 70% in 2018).


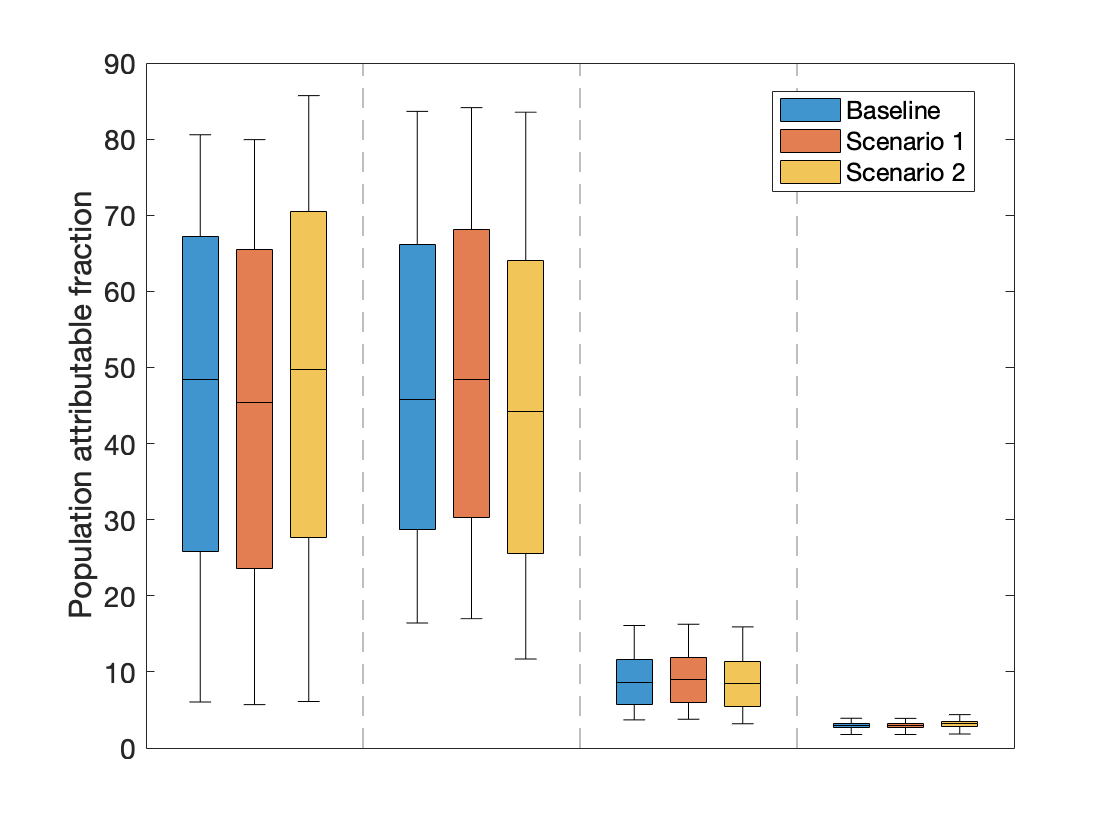


Contribution of different risk behaviours

Unprotected sex between men

Unsafe injecting drug use

Unprotected commercial sex

Unprotected heterosexual main and casual partnerships

Supplementary Figure S10 shows the contribution of different risk behaviours for the Baseline model runs (shown in the main paper) and over 2010-2019 to compare the difference between the time periods.

**Supplementary Figure S10:** The contribution of different risk behaviours to HIV transmission in Tijuana. This is estimated as the percentage decrease in new HIV infections among all key populations when HIV transmission risk due to specific risk behaviours is removed over 2020-2030 in Tijuana. Boxes show the 25^th^, 50^th^ and 75^th^ percentiles and the whiskers indicating the 2.5^th^-97.5^th^ percentiles over the 5,000 baseline model fits. The different risks removed are no injecting HIV transmission risk; no HIV transmission risk due to sex between men; no HIV transmission risk due to commercial sex; and no HIV transmission risk due to heterosexual main and casual partnerships. The scenarios are (1) over 2010-2019 and (2) over 2020-2029.


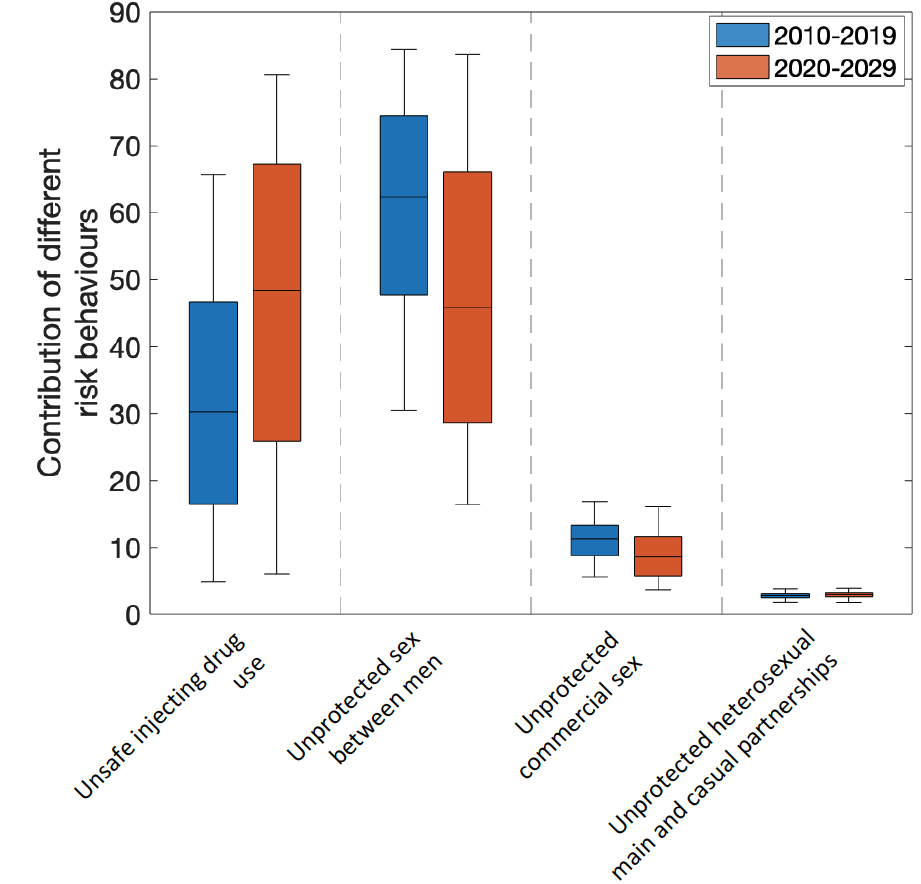


Supplementary Figure S11 shows the contribution of different risk behaviours on for the Baseline model runs (shown in the main paper) and when the model is calibrating to prevalence overall among PWID, among female PWID, overall among FSW, among FSW-PWID and among all MSM.

**Supplementary Figure S11:** The contribution of different risk behaviours to HIV transmission in Tijuana. This is estimated as the percentage decrease in new HIV infections among all key populations when HIV transmission risk due to specific risk behaviours is removed over 2020-2030 in Tijuana. Boxes show the 25^th^, 50^th^ and 75^th^ percentiles and the whiskers indicating the 2.5^th^-97.5^th^ percentiles over the 5,000 baseline model fits. The different risks removed are no injecting HIV transmission risk; no HIV transmission risk due to sex between men; no HIV transmission risk due to commercial sex; and no HIV transmission risk due to heterosexual main and casual partnerships. The scenarios are (1) using the calibration methods described in the main paper and (2) calibrating to additional prevalence data as described above.


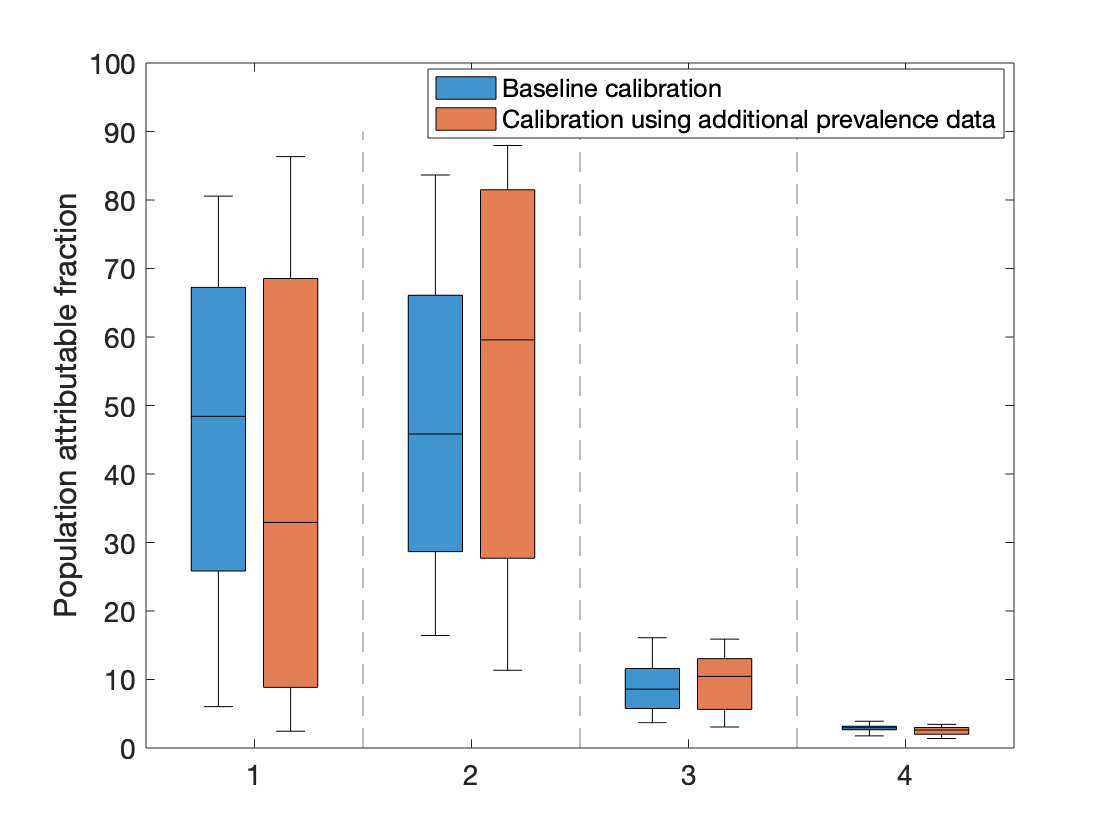


Unprotected heterosexual main and casual partnerships

Unprotected sex between men

Unprotected commercial sex

Unsafe injecting drug use

Contribution of different risk behaviours

# References

1. Iniguez-Stevens E, Brouwer KC, Hogg RS, et al. [Estimating the 2006 prevalence of HIV by gender and risk groups in Tijuana, Mexico]. *Gaceta medica de Mexico* 2009; **145**(3): 189-95.

2. Baggaley RF, White RG, Boily MC. HIV transmission risk through anal intercourse: systematic review, meta-analysis and implications for HIV prevention. *International journal of epidemiology* 2010; **39**(4): 1048-63.

3. Boily MC, Baggaley RF, Wang L, et al. Heterosexual risk of HIV-1 infection per sexual act: systematic review and meta-analysis of observational studies. *The Lancet Infectious diseases* 2009; **9**(2): 118-29.

4. Mukandavire C, Walker J, Schwartz S, et al. Estimating the contribution of key populations towards the spread of HIV in Dakar, Senegal. *Journal of the International AIDS Society* 2018; **21 Suppl 5**: e25126.

5. Hollingsworth TD, Anderson RM, Fraser C. HIV-1 transmission, by stage of infection. *The Journal of infectious diseases* 2008; **198**(5): 687-93.

6. Morgan D, Mahe C, Mayanja B, Whitworth JA. Progression to symptomatic disease in people infected with HIV-1 in rural Uganda: prospective cohort study. *BMJ (Clinical research ed)* 2002; **324**(7331): 193-6.

7. Lawn SD, Little F, Bekker LG, et al. Changing mortality risk associated with CD4 cell response to antiretroviral therapy in South Africa. *AIDS (London, England)* 2009; **23**(3): 335-42.

8. Brinkhof MW, Boulle A, Weigel R, et al. Mortality of HIV-infected patients starting antiretroviral therapy in sub-Saharan Africa: comparison with HIV-unrelated mortality. *PLoS medicine* 2009; **6**(4): e1000066.

9. Mills EJ, Bakanda C, Birungi J, et al. Mortality by baseline CD4 cell count among HIV patients initiating antiretroviral therapy: evidence from a large cohort in Uganda. *AIDS (London, England)* 2011; **25**(6): 851-5.

10. May M, Boulle A, Phiri S, et al. Prognosis of patients with HIV-1 infection starting antiretroviral therapy in sub-Saharan Africa: a collaborative analysis of scale-up programmes. *Lancet (London, England)* 2010; **376**(9739): 449-57.

11. Mocroft A, Kirk O, Aldins P, et al. Loss to follow-up in an international, multicentre observational study. *HIV medicine* 2008; **9**(5): 261-9.

12. Strathdee SA, Lozada R, Martinez G, et al. Social and structural factors associated with HIV infection among female sex workers who inject drugs in the Mexico-US border region. *PloS one* 2011; **6**(4): e19048.

13. Patterson TL, Mausbach B, Lozada R, et al. Efficacy of a brief behavioral intervention to promote condom use among female sex workers in Tijuana and Ciudad Juarez, Mexico. *American journal of public health* 2008; **98**(11): 2051-7.

14. Vera A, Abramovitz D, Lozada R, et al. Mujer Mas Segura (Safer Women): a combination prevention intervention to reduce sexual and injection risks among female sex workers who inject drugs. *BMC public health* 2012; **12**: 653.

15. Strathdee SA, Abramovitz D, Lozada R, et al. Reductions in HIV/STI incidence and sharing of injection equipment among female sex workers who inject drugs: results from a randomized controlled trial. *PloS one* 2013; **8**(6): e65812.

16. Syvertsen JL, Robertson AM, Abramovitz D, et al. Study protocol for the recruitment of female sex workers and their non-commercial partners into couple-based HIV research. *BMC public health* 2012; **12**: 136.

17. Servin AE, Brouwer KC, Gordon L, et al. Vulnerability Factors and Pathways Leading to Underage Entry into Sex Work in two Mexican-US Border Cities. *The journal of applied research on children : informing policy for children at risk* 2015; **6**(1).

18. Conners EE, West BS, Roth AM, et al. Quantitative, Qualitative and Geospatial Methods to Characterize HIV Risk Environments. *PloS one* 2016; **11**(5): e0155693.

19. Patterson TL, Goldenberg S, Gallardo M, et al. Correlates of HIV, sexually transmitted infections, and associated high-risk behaviors among male clients of female sex workers in Tijuana, Mexico. *AIDS (London, England)* 2009; **23**(13): 1765-71.

20. Pitpitan EV, Chavarin CV, Semple SJ, Magis-Rodriguez C, Strathdee SA, Patterson TL. Hombre Seguro (Safe Men): a sexual risk reduction intervention for male clients of female sex workers. *BMC public health* 2014; **14**: 475.

21. Pitpitan EV, Goodman-Meza D, Burgos JL, et al. Prevalence and correlates of HIV among men who have sex with men in Tijuana, Mexico. *Journal of the International AIDS Society* 2015; **18**: 19304.

22. Robertson AM, Garfein RS, Wagner KD, et al. Evaluating the impact of Mexico's drug policy reforms on people who inject drugs in Tijuana, B.C., Mexico, and San Diego, CA, United States: a binational mixed methods research agenda. *Harm reduction journal* 2014; **11**: 4.

23. Cepeda JA, Burgos JL, Kahn JG, et al. Evaluating the impact of global fund withdrawal on needle and syringe provision, cost and use among people who inject drugs in Tijuana, Mexico: a costing analysis. *BMJ open* 2019; **9**(1): e026298.
